# Polarization induced self-doping in epitaxial Pb(Zr_0.20_Ti_0.80_)O_3_ thin films

**DOI:** 10.1038/srep14974

**Published:** 2015-10-08

**Authors:** Lucian Pintilie, Corneliu Ghica, Cristian Mihail Teodorescu, Ioana Pintilie, Cristina Chirila, Iuliana Pasuk, Lucian Trupina, Luminita Hrib, Andra Georgia Boni, Nicoleta Georgiana Apostol, Laura Elena Abramiuc, Raluca Negrea, Mariana Stefan, Daniela Ghica

**Affiliations:** 1 National Institute of Materials Physics, Atomistilor 105 bis, Magurele, 077125, Romania

## Abstract

The compensation of the depolarization field in ferroelectric layers requires the presence of a suitable amount of charges able to follow any variation of the ferroelectric polarization. These can be free carriers or charged defects located in the ferroelectric material or free carriers coming from the electrodes. Here we show that a self-doping phenomenon occurs in epitaxial, tetragonal ferroelectric films of Pb(Zr_0.2_Ti_0.8_)O_3_, consisting in generation of point defects (vacancies) acting as donors/acceptors. These are introducing free carriers that partly compensate the depolarization field occurring in the film. It is found that the concentration of the free carriers introduced by self-doping increases with decreasing the thickness of the ferroelectric layer, reaching values of the order of 10^26^ m^−3^ for 10 nm thick films. One the other hand, microscopic investigations show that, for thicknesses higher than 50 nm, the 2O/(Ti+Zr+Pb) atomic ratio increases with the thickness of the layers. These results suggest that the ratio between the oxygen and cation vacancies varies with the thickness of the layer in such a way that the net free carrier density is sufficient to efficiently compensate the depolarization field and to preserve the outward direction of the polarization.

The key property of ferroelectrics is the presence of the spontaneous polarization, which can be controlled with an external electric field^1^. The result is the well known hysteresis cycle, or *P-E(V)* loop (*P*-polarization; *E*-electric field; *V*-voltage)^2^. Therefore, by applying a suitable external electric field one can align the polarization in the direction parallel to the field, obtaining in this way a mono-domain state. Assuming a capacitor-like geometry, it results that one face of the plate will be positively charged and the opposite one will be negatively charged. The two sheets of surface charges will generate an internal electric field, called depolarization field, oriented in opposite direction to the existing polarization. If there are no electrodes on the opposite faces of the ferroelectric slab, no free charges available in the volume of the ferroelectric material, and no charged species (adsorbants) in the surrounding atmosphere, then the depolarization field will lead to the formation of ferroelectric domains in order to lower the free energy of the system^1^. It results that the mono-domain state can be preserved only if there are available free charges to compensate the depolarization field^3^,^4^. The classical knowledge is that the ferroelectrics are insulators and that the compensating charges are provided only by the metal electrodes deposited on the ferroelectric slab to form a capacitor^5^,^6^,^7^. This assumption is not working for thin films of ferroelectric materials with perovskite structure like Pb(Zr,Ti)O_3_ (PZT) or BaTiO_3_, especially if the films are of epitaxial quality. Many of the recent experimental results (e.g. presence of the Schottky-type contacts with polarization controlled properties, significant values of the leakage current, etc.) show that in the latter case the ferroelectrics have to be considered as (wide) band gap semiconductor with an important density of free (mobile) charges inside de film^8^,^9^,^10^,^11^,^12^,^13^. On the other hand, it has been proven both theoretically and experimentally that the epitaxial films with tetragonal distortion grown on conductive oxide layers, such as SrRuO_3_ (SRO) or (La,Sr)MnO_3_ (LSMO), present a dominant out-of-plane polarization^14^,^15^,^16^. This fact suggests that the concentration of the free carriers during the film growth is sufficiently high to compensate the depolarization field and to preserve a mono-domain state. The free carriers can be supplied by: 1) the bottom electrode; 2) by the film itself through the inherent impurities that are present in the raw materials used to grow the epitaxial films (e.g. the targets used for pulse laser deposition (PLD), or radio-frequency (RF) sputtering), or through the structural defects that occur during the film growth (e.g. vacancies of the component elements); 3) by the environment existent in the deposition chamber, that may contain charged species which are adsorbed on the film surface^17^.

Here we focus on the free carriers which are present in the ferroelectric PZT epitaxial films and we show that their concentration is large and increases with decreasing the film thickness, playing a significant role in the screening of the polarization charges in ultra thin films. We suggest that the screening in ultra thin films is dominantly intrinsic while in thick films is dominantly extrinsic (free carriers from electrodes or adsorbants from the atmosphere). We attribute the presence of a high free carrier concentration in PZT films to a self-doping phenomenon that takes place during the film growth. The self-doping is a process of generating free charges without the intentional introduction of impurities or structural defects acting as donors/acceptors in the studied materials. For example, free carriers can be generated by vacancies, anti-sites or interstitials occurring in the sample during the preparation phase^18^,^19^,^20^,^21^. Self-doping may occur also in order to preserve the local charge neutrality, as is the case for ferroelectric thin films with dominant out-of-plane polarization.

The self-doping hypothesis has emerged observing that, for a set of films of different thicknesses but with the same bottom and top electrodes, the magnitude of the leakage current increases with decreasing the film thickness while the value of the spontaneous polarization remains about the same^22^,^23^,^24^,^25^. The assumption that all the free carriers from the ferroelectric films are involved in the compensation of the depolarization field (preservation of charge neutrality), correlated with the observed increase in the leakage current, lead us to the idea that the concentration of the free carriers has to increase with decreasing the thickness of the ferroelectric layer. Indeed, for the case of PZT films, having a polarization of about 1 Cm^−2^ 26,27, the total free charge density required to fully compensate the depolarization field is calculated to be ~1.25 × 10^27^ m^−3^ for a 5 nm thick film and ~2.5 × 10^25^ m^−3^ for a film of 250 nm thickness. As mentioned above, the free charges may originate from the bottom electrode, from the surrounding atmosphere (if the top electrode is missing), or may be present in the ferroelectric film itself. Considering that the films are not intentionally doped and that the growth atmosphere in the case of PLD or RF-sputtering did not contain organic radicals, it results that the free carriers in ferroelectric are generated by a self-doping mechanism during the growth phase. This hypothesis is supported also by the following facts:

- The PZT films are usually grown on single crystal SrTiO_3_ (STO) substrates with (001) orientation, and with SrRuO_3_ (SRO) bottom electrode. These films are grown at temperatures around 600 °C, in low oxygen pressure. In this respect one should compare the deposition temperature with that of the phase transition in order to know if the PZT film grows in the paraelectric or ferroelectric state. For the selected PZT composition (Zr/Ti ratio of 20/80), in bulk form, the transition temperature is about 450 °C but can be significantly higher in the case of strained thin films, going up to 680 °C^28^,^29^,^30^. It means that the film may grow from the beginning in the ferroelectric phase, with non-zero spontaneous polarization.
- The PZT films on SRO bottom electrode grow with dominant upward polarization (oriented from the bottom SRO electrode to the surface, called UP or P^(+)^ polarization^14^,^31^), suggesting that during the growth there are enough free charges in the film to compensate the depolarization field and to preserve the upward polarization orientation. The free charges can result from point defects occurring during the growth, most probably vacancies of the component elements.

Before presenting the results and discussion one has to underline that the self-doping mechanism, which does not involve any intentional change in the stoichiometry to introduce defects acting as donors or acceptors, cannot be assimilated to the introduction of vacancies by the intentional alteration of the stoichiometry. A previous study has shown that vacancies introduced by changing the stoichiometry in PbTiO_3_ powders may be involved in the intrinsic screening of the depolarization field in thin films^32^. In the present study all the films were deposited from the same target, with no intentional change in stoichiometry. Therefore, the intentional introduction of vacancies acting as sources of free carriers is excluded.

## Results

The self-doping hypothesis presented above was checked by performing complex investigations on a set of epitaxial PZT films with different thicknesses: 5 nm, 10 nm, 20 nm, 50 nm, 100 nm, 150 nm, 200 nm, 250 nm and 300 nm. Samples from these films were used for structural investigations by X-ray diffraction (XRD), atomic force microscopy (AFM) and high resolution transmission electron microscopy (HRTEM), as well as for chemical analysis by X-ray photoelectron spectroscopy (XPS) and scanning TEM (STEM) combined with electron energy loss spectroscopy (EELS) and energy dispersive X-ray spectroscopy (EDS). Piezoelectric force Microscopy (PFM) was also used to investigate the presence of ferroelectric domains in the as-grown films. Other samples from the deposited PZT films were used for macroscopic electrical characterization. Top SRO electrodes were deposited for this purpose (100 × 100 μm^2^).

Before starting any investigations on thin films, the content of impurities in the targets used for PLD growth was investigated using electron paramagnetic resonance (EPR), which is a highly sensitive method for the identification and quantitative evaluation of the paramagnetic species present in a sample. The EPR investigation of a powder sample scratched from the PZT target used for the deposition of the films revealed centers associated with iron and chromium impurities, namely: Fe^3+^ 33, a Fe^3+^- V_O_ (oxygen vacancy) complex^34^ and Cr^5+^ 35. Paramagnetic centers associated with Ti/Zr vacancies seemed to be also present^36^,^37^. However, the density of these centers is close to the detection limit of EPR, below 10^23^ m^−3^. This is at least one order of magnitude lower than previously reported carrier concentrations^11^,^12^,^13^. In order to check the transfer of impurities from the target to the film, we have investigated a PZT layer of 1 μm thickness, deposited on a highly resistive floating zone Si(001) substrate with a 40 nm buffer layer of SrTiO_3_. Even in this thicker film no EPR spectra from paramagnetic impurities or intrinsic defects could be observed (details on the EPR investigations can be found in Supplemental Information-SI). One can conclude that the amount of impurities acting as donor or acceptor centers and coming from the deposition target is well below 10^23^ m^−3^, far too low to explain the large leakage current and the efficient compensation of the out-of-plane polarization in epitaxial PZT films. Therefore, point defects acting as dopants have to be generated during the PZT growth. Free carriers are introduced in this way, which are involved in the partial compensation of the depolarization field established perpendicular to the substrate (and to the growth surface) due to the presence of the out-of-plane polarization.

### Structural investigations

**Figure 1:**
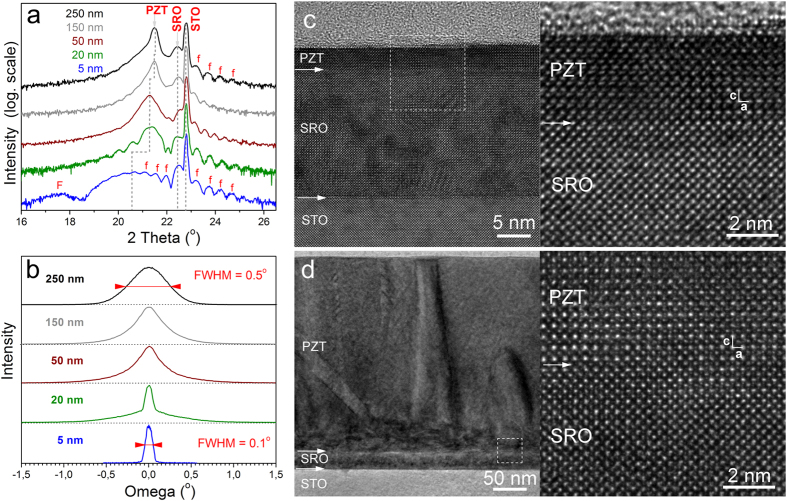
(**a**) Details of the *2θ−ω* scans around the (001) diffraction peaks of STO and of the pseudocubic structures—the diagrams are shifted vertically; (**b**) rocking curves of PZT taken with the (002) reflection—curves presented with normalized heights and shifted vertically; (**c**) low-magnification and HRTEM images of the 5 nm thick PZT film; (**d**) low-magnification and HRTEM images of the 250 nm thick PZT film.

The results of the structural investigations performed on the epitaxial PZT films with different thicknesses are shown in Fig. 1. The XRD analysis demonstrated the epitaxial growth of the SRO and PZT films. The *2θ-ω* scans presented in (Fig. 1a) reveal in all samples the layer fringes (marked with “f”) associated to the thin SRO buffer layer indicating the atomic scale smoothness of the interfaces, and the uniform thickness (~20 nm from the layer fringes period) of this layer. Also a few fringes of the thinner PZT layer were evidenced (marked with “F” in the figure), proving that the layer is very smooth and has a uniform thickness of 4–5 nm as estimated from the fringes period. The diagrams also evidence that the out-of-plane lattice parameter of the thinnest PZT film is significantly larger (*c*_5 nm_ = 4.230 Å) than that of 20 nm or thicker ones (*c*_20 nm_ = 4.160 Å, *c*_250 nm_ = 4.135 Å ≈ *c*_bulk_ = 4.132 Å—according to ICDD # 70–4260), suggesting that the 20 nm thick PZT layer begins to relax, and the relaxation proceeds slowly until the greatest thickness. The XRD analysis revealed also that, except the ultra-thin films (below 20 nm), the other films seem to be composed of two types of PZT: a strained layer at the interface with the SRO electrode, with an in-plane lattice constant of about 3.92 ± 0.01 Å for all thicknesses (one has to notice that this value is very close to the in-plane lattice constant of the SRO layer, which is 3.928 Å); a relaxed layer, with an in-plane lattice constant varying from 3.955 Å for 50 nm thickness to 3.985 Å for 250 nm thickness. These results are similar to other reports^38^ and suggest a change in the growth mechanism from 2D layer-by-layer growth (Frank-van der Merwe) to mixed growth, first layer-by-layer and then 3D island growth (Stranski-Krastanov).

The rocking curves presented in (Fig. 1b) show that the 5 nm PZT film grows like as a single crystal on the SRO bottom electrode. For the other thicknesses the shape of the rocking curve changes and the widths increase suggesting the occurrence of misfit dislocations, and other defects which alter the out-of-plane crystal orientation. This is confirmed by the low-magnification TEM images presented in (Fig. 1c,d). One can see that for the 250 nm thick film strain contrast is associated to the presence of grain boundaries and a high density of dislocations, especially at the PZT-SRO interface. Other extended defects such as threading dislocations and 90° domains are also present in the volume of the PZT layer, as seen in (Fig. 1d) for low magnification. TEM investigations revealed that the PZT films start to relax forming dislocations and domains from a thickness of 50 nm (see Figs S4 to S8 in SI). The relaxation of the compressive stress exerted by the STO substrate (*a* = 3.905 Å) onto the PZT layer with increasing the PZT thickness has been also evidenced by measuring the lattice parameters on the HRTEM micrographs. Thus, a PZT lattice parameter *c* = 4.21 Å has been measured in the 5 nm layer compared with *c* = 4.12 Å measured in the 250 nm thick layer, which is in perfect agreement with the XRD data. The structural investigations are thus confirming that the films are of good epitaxial quality.

**Figure 2:**
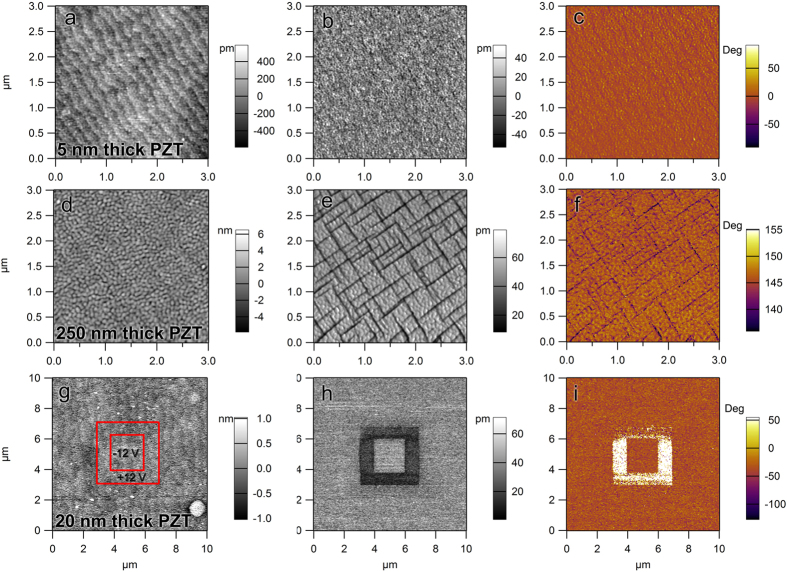
(**a–c**) AFM topography, PFM amplitude and phase for the 5 nm thick PZT film—unit cell step terraces can be observed; (**d–f**) same for the 250 nm thick film—the characteristic grid of 90^0^ domains can be observed; (**g–i**) poling map, PFM amplitude and phase for a 20 nm thick film—one can observe that the inner square, poled UP (P^+^), has the same phase contrast as the unpoled area outside the outer square.

The presence of ferroelectric domains in thicker films is confirmed by the AFM-PFM images shown in Fig. 2. One can observe, in the AFM topography image (Fig. 2a), that the surface morphology of the 5 nm thick PZT thin film still follows the step terraces of the SRO layer. The phase and amplitude images of the piezoresponse signal (Fig. 2b,c) shows no variations in contrast, suggesting the presence of the mono-domain state (large regions with out-of-plane oriented polarization). For the 250 nm thick film the topography as well as the amplitude and phase of the piezoresponse signal present the characteristic grid of 90° domains (see Fig. 2d–f). Thus, only the thickest films show that part of the volume has in-plane polarization. The percent of 90° domains, with in-plane polarization, was estimated to be about 15% for the 250 nm thick film.

The AFM-PFM analysis proves the dominance of the P^(+)^ polarization, as suggested by previous studies^14^,^31^. This fact was evidenced by using the following procedure: 1) local areas were poled up and down; 2) phase contrast of the poled areas was then compared with the contrast of the unpoled ones; 3) if there is no contrast difference between the poled and unpoled areas it means that the polarization has the same orientation in the two areas. This orientation was found to be P^(+)^ (see Fig. 2g–i).

### Electric measurements

**Figure 3:**
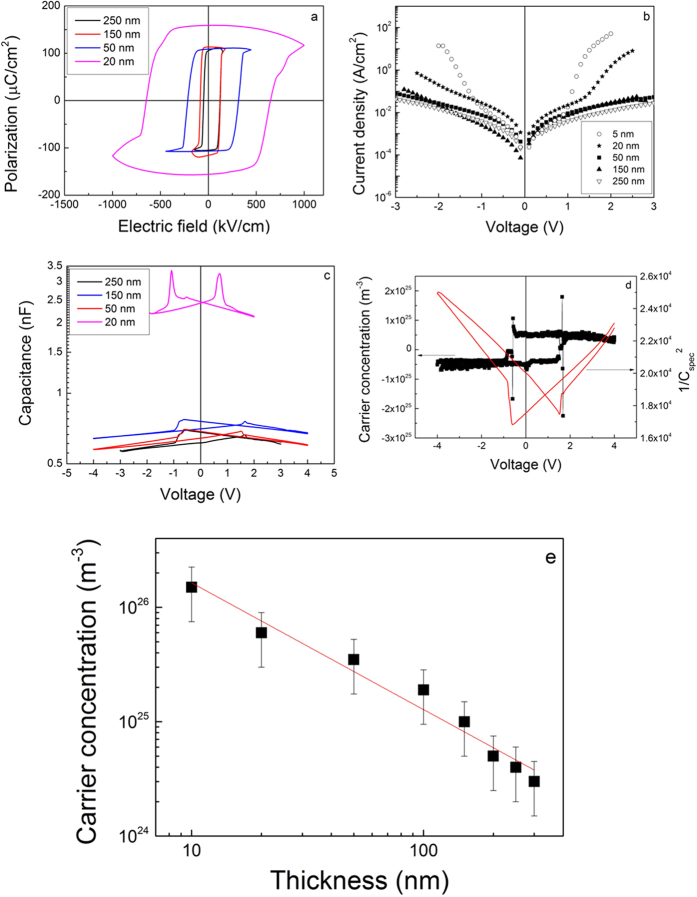
PZT samples of different thicknesses. (**a**) hysteresis loops; (**b**) I-V characteristics; (**c**) C-V characteristics; (**d**) example for estimation of free carrier concentration (details in SI); (**e**) the thickness dependence of the free carrier concentration as determined from C-V characteristics.

The hysteresis measurements performed on the films with different thicknesses have revealed that the hysteresis loops start to be inflated by the leakage current as the thickness decreases (see for example the 20 nm thick film in Fig. 3a). The polarization values were found to be about the same down to 10 nm thickness, of 1 ± 0.05 C/m^2^ (see Fig. 3a). The contribution of the leakage current was extracted following a procedure that is described in SI for the film with 10 nm thickness. For the 5 nm thick film it was not possible to obtain a meaningful hysteresis loop due to the very large leakage current. The current-voltage (I-V) characteristics have also confirmed a significant increase of the leakage current for very low thicknesses, especially for the 5 nm thick film for which the value of the current at 2 V is with more than two orders of magnitude higher than for the 250 nm one (see Fig. 3b). The capacitance-voltage (C-V) characteristics (examples are presented in Fig. 3c) were recorded at room temperature. One has to notice that the capacitance at +/−2 V follows the 1/thickness dependence within an error of about 5% originating mainly from the different densities of structural defects which can add (or not) extrinsic contributions to the measured capacitance. The data were then used to extract the concentration *n* of the free carriers using the same procedure as in the case of the metal-semiconductor Schottky contacts^39^,^40^. An example is presented in (Fig. 3d) for a 200 nm thick film. The dielectric constant used in calculations was the one extracted from the C-V characteristic at the highest applied voltage, where the contribution from the polarization switching is negligible^13^. The estimated free carrier concentrations are represented in (Fig. 3e) as a function of thickness in log-log scale.

One can see that the dependence is linear, with a confidence factor of 98.5% and a slope of −1.1. The intercept is around 2 × 10^27^ m^−3^. This result leads to the following empirical dependence of the free carrier concentration on the thickness *d* of the PZT film:


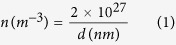


with thickness *d* expressed in nm, and concentration *n* obtained in m^−3^. It results that for a film of about 1 nm thickness, which appears to be the limit for the presence of polarization^41^, the free carrier concentration should be ~2 × 10^27^ m^−3^. The value is similar with the one predicted by theoretical calculations in BaTiO_3_^42^. For a 100 nm thick film the free carrier concentration is about 2 × 10^25^ m^−3^.

The first observation is that the concentration of the free carriers in epitaxial PZT films is with orders of magnitude larger than the density of possible donor/acceptor defects determined by EPR. The second observation is that the same target was used to grow all the films, underlining that the target was not intentionally doped except the 10% surplus of PbO added to compensate the PbO losses during the target sintering process. Therefore, no intentional change in stoichiometry was made to introduce cation or oxygen vacancies like in Ref. 32. Considering that the deposition parameters were the same, one can explain the increased density of free carriers with decreasing the thickness of the PZT layer by self-doping mechanism introducing the required free charges to efficiently compensate the dominant outward direction of polarization evidenced by PFM studies (see Fig. 2). One can estimate the amount of polarization charge that can be compensated with free carriers from the PZT layers of different thicknesses. It results that for very thin films, of 5 to 10 nm, up to 50% of the polarization is compensated by the free carriers from the ferroelectric layer, while for the films of 250–300 nm the percentage drops to about 10%. One can assume that in thicker films the extended defects observed in TEM images (see Fig. 1d) act as efficient trapping centers for the free carriers^43^. The result will be a reduction of the free carrier concentration, thus the contribution of the carriers coming from the bottom SRO electrode to the compensation process of the depolarization field will increase with the PZT thickness. From the above results one can conclude that for the very thick films the concentration of the free carriers in the PZT layer becomes too small to efficiently contribute to the compensation of the depolarization field and thus, one can consider that the entire compensation process takes place at the electrode interfaces, with carriers from the electrodes. These findings show that the compensation mechanisms are different in bulk samples or thick films compared with the very thin layers and support the statement that PZT can be considered an insulator if the layer is thick enough to neglect the concentration of the free carriers resulted from the self-doping phenomenon that occurs during the growth of the film. Otherwise, it has to be considered as a wide band gap semiconductor with a significant concentration of free carriers. However, the nature of the defect(s) introduced into the PZT layer by self-doping, and responsible for the large density of free carriers, is not known. It can be assumed that these are vacancies.

### TEM and XPS investigations

**Figure 4:**
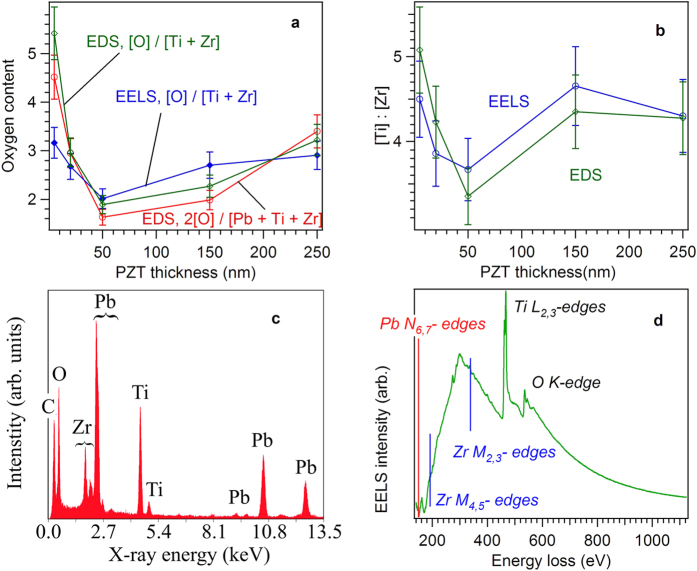
(**a**) 2O/(Ti+Zr+Pb) atomic ratio (TEM-EDS measurements) and O/(Ti+Zr) atomic ratio (TEM-EDS and EELS measurements) versus the PZT layer thickness (lines are used as guide for the eye); (**b**) Ti/Zr ratio obtained by EELS and EDS, as function of PZT film thickness; (**c**) Typical EDX spectrum of the analyzed PbZr_0.2_Ti_0.8_O_3-x_ layers. (**c**) Typical EEL spectrum of the analyzed PbZr_0.2_Ti_0.8_O_3-x_ layers, after background removal, showing the absorption edges Zr M, Ti L and O K.

To gain more insight on this problem, the PZT thin films stoichiometry has been investigated by TEM-EDS and EELS techniques. EDS and EELS spectra have been acquired on the cross-section TEM specimens prepared from the 5 nm, 20 nm, 50 nm, 150 nm and 250 nm thick samples. In order to reduce the errors in quantifying the oxygen in the EDS spectra, only the thinnest areas of the cross-section specimens have been investigated. The quantitative data has been averaged on several spectra acquired in neighboring thin areas. The 2O/(Ti+Zr+Pb) atomic ratio has been calculated from EDS spectra and plotted in (Fig. 4a) as a function of the thickness of the PZT layer. For the quantitative analysis of the EDS spectra, the K lines have been considered for O, Ti and Zr, while for Pb the M line has been used (see Fig. 4c). The obtained values, including a 10% error margin, are distributed around the stoichiometric atomic ratio of 3. The graph in (Fig. 4a) indicates an oxygen deficit for the 50 nm and 150 nm PZT layers, with a clear trend of increase in the oxygen content towards the stoichiometric value for the 250 nm layer. The stoichiometric value has been noticed for the thin PZT layer of 20 nm. However, a ratio value higher than the stoichiometric one has been estimated for the 5 nm layer, indicating some cationic deficit.

EDS and EELS analysis were then used to evaluate the O/(Ti+Zr) atomic ratio (Fig. 4a). The EELS spectra (see an example in Fig. 4d) were recorded in diffraction mode (image coupled) and the quantification was performed after removing the background using a power law model. The plots of the O/(Ti+Zr) atomic ratios as function of PZT thickness, considering a 10% error margin, indicates a similar trend as the one obtained from the EDS analysis for the 2O/(Ti+Zr+Pb) ratio. The O/(Ti+Zr) value is over the stoichiometric limit of 3 for the 5 nm thick film, indicating cationic deficit. For the 20 nm thick film the ratio is near 3, and drops below 3 at 50 nm with the tendency to increase towards the stoichiometric value as the thickness increases to 250 nm. All the above results indicate that the oxygen/cation ratio increases as the thickness increases from 50 nm to 250 nm. For thicknesses below 50 nm the EDS and EELS results indicate cationic deficit. One has to mention that the measured composition values can be affected by significant errors, especially in the case of O measured by EDS, where a slight overvaluation is not excluded. This is due to inherent unknowns such as precise specimen thickness (crossed by the electron beam), specimen-detector geometry, or selected ionization line (M line for Pb, K line for the rest of the elements). However, in our measurements we have tried to preserve identical measurement conditions in order to evidence the trend of the chemical composition with respect to the thickness of the PZT layer.

The Ti/Zr ratio was also estimated from EDS and EELS and was found to be around the expected value of 4, considering that the chemical formula is Pb(Zr_0.2_Ti_0.8_)O_3_ (see Fig. 4b).

The variation of the oxygen/cation ratio with the film thickness, as shown in (Fig. 4a), is possible if oxygen and/or cation vacancies are generated. V_O_ can introduce free electrons (donor) while V_cat_ can introduce free holes (acceptor)^44^,^45^,^46^. Both electrons and holes can contribute to the compensation of the polarization charges generating the depolarization field. However, the free carrier concentration estimated from the C-V measurements and presented in Fig. 3e is a difference between the concentrations of the two types of charge carriers. It results that either the donor concentration V_O_ or the acceptor concentration V_cat_ is dominant and is responsible for the free carrier concentration determined from the electrical measurements. Recent theoretical studies have shown that the V_O_ is the most favorable defect to occur in PbTiO_3_ grown in oxygen-poor conditions^44^. Considering that the PLD growth of the present PLD films takes place in an oxygen pressure of only 0.2 mbar one can consider that the oxygen-poor conditions are fulfilled and that the probable defect to form is V_O_. Therefore, one can expect that the 2O/(Pb+Ti+Zr) ratio is below the stoichiometric value of 3, as suggested by the results of TEM analysis for samples thicker than 50 nm. The higher oxygen content in thinner films will be discussed later on, in relation with the XPS results.

**Figure 5:**
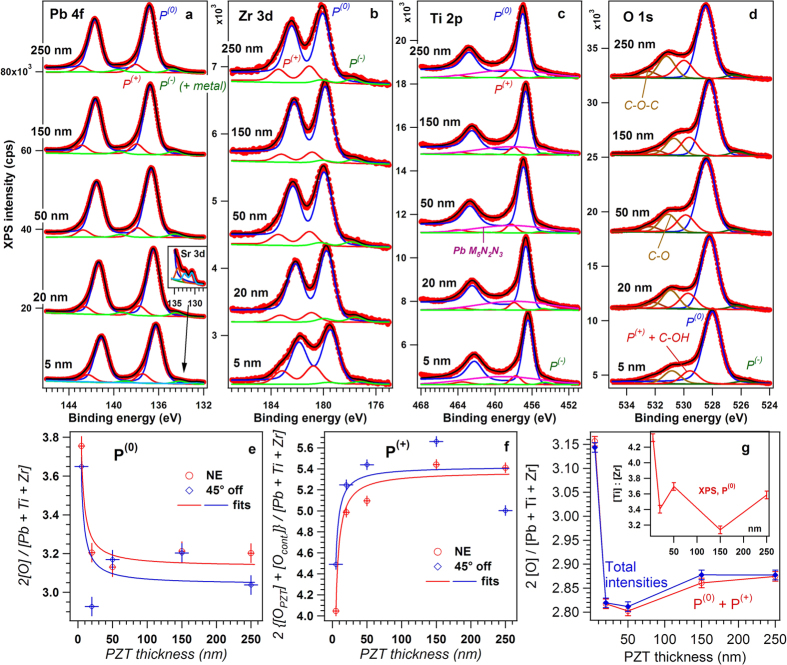
X-ray photoelectron spectroscopy (XPS) results for PZT layers of variable thicknesses: 5, 20, 50, 150, and 250 nm. (**a**) Pb 4 f, (**b**) Zr 3d, (**c**) Ti 2p, (**d**) O 1 s. Spectra are deconvoluted into three components, corresponding to in-plane polarization P^(0)^ and out-of-plane polarizations P^(±)^ (see text for details), with the exception of O 1 s, where two additional components due to inherent contamination are introduced. For Ti 2p, a broad singlet+background simulating a Pb Auger line was introduced. (**e,f**) represent the evolution with the layer thickness of the oxygen content derived individually for components with well defined polarization P^(0)^ for (**e**) and P^(+)^ for (**f**), together with fits using formula (6). (**g**) Represents total ratios of oxygen/cations for the total spectra (blue curve) and for the sum between P^(0)^ and P^(+)^ components (red curve).

The results of the XPS investigations are presented in Fig. 5a–d, as series of spectra (Pb 4f, Zr 3d, Ti 2p and O 1s) obtained for all PZT thicknesses investigated, at normal emission (NE). Spectra were recorded also at oblique incidence (45° take-off angle, for increased surface sensitivity; they are presented in SI, but the results obtained will be included in the discussion below). The measurements were performed without making any special surface treatment for contaminants removal, in order to avoid possible alteration of the composition in the surface layer (e.g. loss of PbO). Therefore, C 1s was visible in the spectra and this was used for calibration to the “adventitious carbon” line at 284.6 eV. The C 1s spectra are given in the SI. Due to this fact and also to the hole generation near the interface by the photoemission process, the relative percentage of areas with different polarizations might be altered with respect to the investigations under air, with no X-ray flux nor electron flux directed towards the sample. Therefore, the ratio between the areas with out-of-plane and in-plane polarizations can be significantly different in the XPS compared to PFM results.

The XPS data are fitted with Voigt line shapes and inelastic backgrounds^47^ (singlets for O 1s and C 1s, doublets with well defined branching ratios (1+1/*l*, *l* being the orbital quantum number for Pb 4f, Zr 3d and Ti 2p). The gaussian line width, connected to the experimental resolution, was kept the same along one spectrum, whereas the lorentzian line width, connected to the X-ray natural width and to the inherent width of the photoemission line, was allowed to vary for Ti 2p only (from the 2p_3/2_ lines to the 2p_1/2_ lines) owing to additional Coster-Kronig decay channels of the core hole opened for the line with the highest binding energy^48^. These additional decay channels do not manifest when the spin-orbit splitting is lower than the work function of the material, as is the case of Pb 4f and Zr 3d. The general strategy of curve fitting was to use the minimum number of components, but it could not be avoided to use four components for the Pb 4f spectra (one component representing the Sr 3d doublet at about 131–133 eV, see the insert of Fig. 5a, three components for the Zr 3d spectra, three doublets plus one broad singlet for the Ti 2p spectra (the singlet corresponding to a Pb Auger line^49^), five singlets for the O 1s spectra, two of these components corresponding to contaminants^50^. If one removes the additional lines whose origin was briefly explained above, all spectral features associated to PZT may be deconvoluted with 3 components (red, blue and green curves from Fig. 5a–d), one main component of largest intensity and two additional components at higher and lower binding energies by roughly the same amount for all spectra and all samples, 2.04 ± 0.12 eV for the component towards lower binding energies, and 1.28 ± 0.15 eV for the component towards higher binding energies.

The natural interpretation of these three components is that the main component, of highest intensity, belongs to areas with no surface band bending, which will be denoted as P^(0)^ in the following, whereas the other two components represent P^(+)^ states (shifted towards higher binding energies) and P^(−)^ states (shifted towards lower binding energies). Despite the fact that PFM revealed dominant P^(+)^ polarization for all samples investigated, for samples analyzed under ultrahigh vacuum and subject to X-ray flux and electron flood gun, the charge carrier dynamics near surface (especially the holes induced by the photoemission process) may alter the relative weight of P^(+)^ and P^(0)^ areas, introducing also areas with P^(−)^ orientation. Indeed, it was recently demonstrated that under an intense X-ray flux serious modifications of the film, starting with its polarization state and ending with reduction processes at its surface may occur due to photogenerated holes near the surface^51^. In the actual case, the X-ray flux (photons/unit area/s) is considerably lower (by about eight orders of magnitude) than in the above Reference, but one cannot preclude that some switching of the surface polarization state might occur. Another possible explanation for the P^(0)^ component derived by XPS is that some signal coming from the bulk of the sample, where bands are not bended, could contribute to this signal, especially when the length parameter of the surface band bending, *δ*, approaches the inelastic mean free path of the photoelectrons (12–16 Å for the core levels investigated here, with Al K_α_^43^).

The next step was to compute separately the compositions for the XPS-derived P^(0)^ and P^(+)^ components from the integral amplitudes weighted by the atomic sensitivity factors^49^,^52^. The 2O/(Pb+Zr+Ti) ratios for areas with P^(0)^ and P^(+)^ polarization orientations were estimated for each thickness of the PZT layer and are presented in Fig. 5e,f. Finally, a global 2O/(Ti+Zr+Pb) ratio was evaluated for the entire surface of the PZT film using the areas with P^(0)^ and P^(+)^ polarization as weighting factors. The result is graphically presented in Fig. 5g. The 2O/(Ti+Zr+Pb) ratio was also estimated including the areas of P^(−)^ polarization and presented in Fig. 5g. It can be seen that the P^(−)^ contribution is very small and can be neglected in the further discussion relating the self-doping with the dominance of P^(+)^ orientation (as revealed by PFM result present in Fig. 2). The trend in Fig. 5g is similar with the one obtained from TEM investigations and presented in Fig. 4a. For very thin layers the oxygen content is a little bit above 3 then drops below 3 at 50 nm, after which it has the tendency to increase towards 3 as the thickness increases.

The Ti/Zr ratio was also estimated and represented in the inset of Fig. 5g. XPS results indicate a slight Ti deficit at the surface, suggesting a composition in between Pb(Zr_0.2_Ti_0.8_)O_3_ and Pb(Zr_0.25_Ti_0.75_)O_3_.

## Discussions

To reconcile the TEM and XPS results, showing a minimum of the 2O/(Ti+Zr+Pb) ratio at a thickness of 50 nm, with the results of the electric measurements showing a monotonic increase in the density of the free carriers with decreasing the thickness, in the light of the self-doping hypothesis, one has to remind that Sr diffusion over a thickness of about 10 nm was reported in the case of PZT films of the same composition as in the present study^53^. As mentioned above, Sr is visible in the XPS spectrum of the 5 nm thick film (see Fig. 5a). Similar thicknesses of Sr contamination are expected also in the thicker films of the present study. Sr can substitute Pb^53^ but part of it (up to 10%) can substitute also Ti/Zr^54^. In the last case it promotes formation of Pb vacancies, in which case the 2O/(Ti+Zr+Pb) ratio will increase, possibly exceeding the stoichiometric value of 3. This seems to be the case for the film of 5 nm thickness, as shown by the by the TEM results presented in Fig. 4a and by the XPS results presented in Fig. 5g. It appears that in this film the density of Pb vacancies (V_Pb_) is significant, and is larger than the density of V_O_. Therefore, it appears that in the thinnest films, below 20 nm, the holes are the dominant charge carriers contributing to the compensation of the bound polarization charges. Based on the presented TEM and XPS results one can conclude that:

- At thicknesses below 50 nm the doping with V_Pb_ acceptors is dominant, due to Sr substitutions on Ti sites, generating a high concentration of holes which preserve the dominant P^(+)^ orientation of polarization.
- Starting with 50 nm thickness the self-doping through generation of V_O_ donors is dominant, again preserving P^(+)^ orientation.

Only from macroscopic C-V measurement it is not possible to discern which type of carriers, electrons or holes, are dominant at each thickness. The important aspect in this case is that the resulting density of free carriers, as difference between the electron and holes concentration, has a clear thickness dependence as shown in Fig. 3e.

A simple model can be developed to explain the self-doping by V_O_ and the thickness dependence observed in TEM and XPS for the oxygen content for PZT films thicker than 50 nm. The charges needed for compensation of the depolarization field may be expressed by a charge surface density of *σ*_*p*_ = 2*P*/*e*. These charges are produced by creation of oxygen vacancies and by injection from the bottom electrode (see Fig. 5g). The former density of carriers may be expressed as:


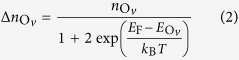


and thus the surface density:


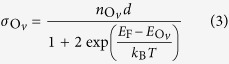


where one has to keep in mind that it may happen that only a percentage of the oxygen vacancies (their density is 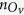) may become ionized and produce free electrons, according to (3), depending on the temperature *T* and on the position of the donor levels 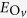 with respect to the Fermi level *E*_F_. The surface density of electrons injected from the metal may be expressed as:


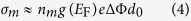


where *n*_*m*_ is the carrier density in the metal, *g*(*E*_F_) is the density of states at the Fermi level, normalized such that 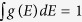, *d*_0_ is the thickness of the metal (SRO), and *e*ΔΦ is the work function difference between PZT and SRO.

From the equality 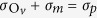 it follows that the density of oxygen vacancies may be expressed as:


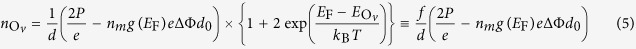


where *f* stands for the statistical factor {1+2 exp(…)}. The oxygen content per formula unit is expressed as:


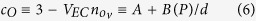


where *V*_*EC*_ is the volume of the elementary cell. The fitting parameters are *A* = 3, whereas *B*(*P*) is expressed as:


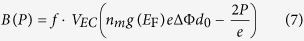


It follows also that:


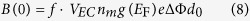


and


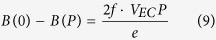


One can see that equation (6) is very similar with equation (1). Both predict a thickness dependence as *1/d*. The difference is the constant term *A* from equation (6), which in the ideal case should be 3. This happens for very large thicknesses, when the density of the oxygen vacancies acting as donors is very much reduced leading to negligible concentration of free carriers, in agreement with equation (1). For small thicknesses the coefficient *B* becomes important.

The two coefficients, *A* and *B*, were determined by fitting equation (6) with the experimental results obtained by XPS for areas with P^(0)^ and P^(+)^ polarizations (see Fig. 5e,f). One obtains for the P^(0)^ component *A* = 3.09 ± 0.05, so fairly close to 3. For the P^(+)^ component *A* = 5.40 ± 0.02, but the oxygen excess in this case can be attributed to contaminants, considering that the O 1s line is superposed on the line usually attributed to C-OH bonds^50^. Actually, the fact that polar contaminant molecules are adsorbed preferentially on P^(+)^ areas was recently observed^52^ and the above finding reinforces this hypothesis. Here it is assumed that the amount of contamination per surface unit cell is roughly the same, irrespective on the thickness of the layer. This was confirmed by the analysis of the C 1s lines (see the details in SI), showing that indeed the amount of carbon is roughly the same on all samples. It is then reasonable to suppose that the O 1s signal originating from C-OH bonds is the same for all samples. From the fitting, one obtains *B*(0) = 2.89 ± 0.11 (nm), *B*(*P*) = −5.65 ± 1.11 (nm). Thus, *B*(0) − *B*(*P*) = 8.54 ± 1.22 (nm). For a polarization of *P* = 1 Cm^−2^ and *V*_*EC*_ ≈ 6.4 × 10^−29^ m^3^, using eq. (8) the factor 
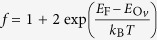
 yields about 10.7 and then, taking into account that *k*_B_*T* ≈ 25 meV, it follows 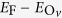 ≈ 0.039 eV. It results that only about 10% of the oxygen vacancies are ionized, but their number is needed in order to achieve statistically the required concentration of electrons via eq. (2). Also, from *B*(0) ≈ 2.89 nm, using eq. (8), and introducing the factor *f* derived above, *n*_*m*_ ≈ 2 × 10^28^ m^−3^ 55, *d*_0_ = 20 nm (thickness of SRO), one may derive the product *g*(*E*_F_)*e*ΔΦ ≈ 0.01. With ΔΦ ≈ 0.25–0.5 Volts^12^, it follows *g*(*E*_F_) ≈ 0.02–0.04 eV^−1^, which is reasonable if one looks at the experimental UPS spectra from SRO^56^.

**Figure 6:**
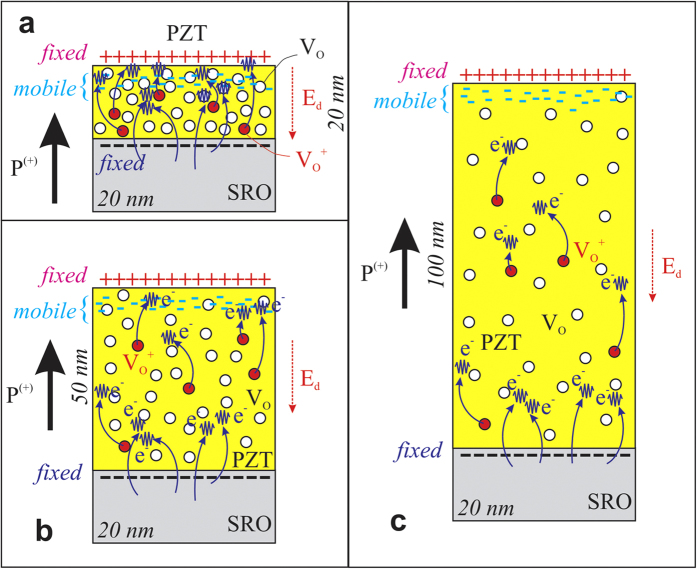
Electrostatic model of a layer with P^(+)^ polarization, where the depolarization field *E*_*d*_ due to the fixed charges at interfaces is compensated by electron accumulation near the surface, and these carriers are produced by the bottom electrode (extrinsic) and by a relatively small proportion of ionized impurities from a high amount of oxygen vacancies (intrinsic). Three thicknesses were schematized: (**a**) 20 nm, (**b**) 50 nm, and (**c**) 100 nm, in order to evidence the decrease in concentration of oxygen vacancies with the increasing layer thickness.

The above presented model predicts that not all oxygen vacancies contribute to the creation of compensation free charges. One can presume that most of them form complexes with other atoms (e.g. Pb-O vacancy pair, or V_O_-Ti^3+^)^57^,^58^,^59^. For the thinnest films there is a competition between forming V_O_ by self-doping and V_Pb_ by Sr substitutions on Ti sites. Taking the free carrier concentration obtained with equation (1) by extrapolation to the lowest thickness where the ferroelectricity still exists (~1 nm), which is about 2 × 10^27^ m^−3^, one can estimate that the V_Pb_ − V_O_ difference should be, roughly, of about 0.05 per unit cell for the thinnest films. It was assumed that each donor/acceptor is double ionized, giving two electrons or two holes. The resulting composition would be of the form Pb_0.9_(Zr_0.2_Ti_0.8_)O_2.95_, which will lead to a 2O/(Ti+Zr+Pb) ratio of about 3.10. The agreement with the result obtained for 5 nm thick film and presented in Fig. 4b is acceptable, considering the errors in XPS evaluations and the assumption that each donor/acceptor is double ionized (single ionized donors/acceptors will require a higher concentration of V_Pb_ or V_O_). The present results are in agreement with other reports claiming the involvement of vacancies in the screening process^60^. However, what underlines the present study is the fact that the vacancies acting as donors/acceptors are introduced by a self-doping mechanism. This is necessary to preserve the dominant outward (P^(+)^) direction of polarization, with the same value of polarization for films with different thicknesses. This is finally reflected in a concentration of free carriers that increases with decreasing the thickness of the PZT layer. As already pointed in the “Electric measurements” paragraph, the screening of the depolarization field in ultra-thin films (below 20 nm) is achieved with equal contributions from the free carriers generated by self-doping in the PZT layer (intrinsic contribution) and electrons coming from the SRO electrode (extrinsic contribution). As the thickness increases, the intrinsic contribution decreases because the concentration of the free carriers decreases. This change is schematically represented in Fig. 6.

The present findings can explain a series of results reported for epitaxial PZT layers such as: large remnant polarization; almost rectangular hysteresis loops; giant pyroelectric coefficient; large densities for the leakage current, increasing with decreasing the thickness. Further studies are needed to learn how to control the self-doping in order to preserve desired orientations and values for the polarization without increasing the leakage current. Polarization control by self-doping during the growth of epitaxial PZT films can be a key element in designing future ferroelectric-based structures for applications in non-volatile memories and photovoltaic cells.

## Methods

### Growth of the films

The films were grown by pulse laser deposition (PLD) method on single crystal, (001) oriented SrTiO_3_ substrates using a KrF (248 nm wavelength) excimer laser. The deposition conditions were as follows: for the SRO bottom electrode-the substrate temperature was 700 °C, the repetition rate 5 Hz, the fluence 2 Jcm^−2^, and the oxygen pressure 0.13 mbar; for the PZT layer- the substrate temperature was 575 °C, the repetition rate 5 Hz, the fluence 2 Jcm^−2^, and the oxygen pressure 0.2 mbar. The substrate temperature during the growth was monitored with a type K thermocouple inserted in the sample holder just beneath the substrate. The as-grown films were post-deposition annealed, in the deposition chamber, at 575 °C, for one hour, in full oxygen atmosphere. Top SRO electrodes of 100 × 100 μm^2^ area were deposited for electrical measurements.

### Electron paramagnetic resonance (EPR) measurements

X (9.8 GHz)- and Q (34 GHz)- band EPR measurements were performed at variable temperature (295–20 K) with the Bruker ELEXSYS-E580 and −E500Q spectrometers from the Center for advanced ESR/EPR techniques (CetRESav). The equipment and magnetic field calibration procedures are described in ref. 61 and at http://cetresav.infim.ro/. The sample holders were quartz tubes of 2 mm and 3 mm inner diameter for the Q-band and X-band measurements, respectively. The EPR spectra were recorded with 100 kHz modulation frequency and relatively high modulation amplitude of 3 G, at the highest microwave power level for which saturation effects did not occur (10 mW). Multiple scans (up to 15) were performed in order to increase the signal to noise ratio.

### Structural characterization

The crystal structure and epitaxial relationships were investigated by high resolution X-ray diffraction (HR-XRD) using a Bruker D8 Advance diffractometer with parallel beam, obtained with multilayer optic (Göbel mirror). The measurements were performed in coplanar geometry with horizontal sample stage, using monochromatized Cu–K_α1_ radiation (*λ* = 1.5406 Å). High-resolution transmission electron microscopy (HR-TEM) analysis was also performed to evaluate the epitaxial quality of the films deposited on STO substrate and to investigate the electrode-ferroelectric interfaces at microscopic level. The investigations were performed with a Cs probe-corrected JEM-ARM 200F electron microscope. The cross-section TEM specimens have been prepared by mechanical grinding and ion milling on a Gatan PIPS machine. The EELS spectra were recorded in diffraction mode (image coupled) using the following experimental conditions: ~0.5 mrad convergence angle (condenser aperture of 150 μm), 1.5 mrad collection angle (60 cm camera length, 2.5 mm spectrometer entrance aperture), 40 seconds total integration time (20 spectra summed).

### XPS investigations

X-ray photoelectron spectroscopy was performed in an analysis chamber (Specs GmbH, Germany) by using a monochromatized Al K_α1_ X-ray source (1486.74 eV) with 350 W anode power. Electrons are analyzed by a 150 mm radius Phoibos electron analyzer operating in large area mode with pass energy of 30 eV, in normal emission. The estimated overall energy resolution (Ag 3d_5/2_ level on a sputter-annealed foil) in these conditions is of ~0.85 eV total full width at half maximum (FWHM), including the experimental broadening of the energy analyzer and core hole lifetimes. A flood gun with acceleration voltage of 1 eV and electron current of 0.1 mA was employed to ensure the sample neutralization. Several test experiments were performed before starting the real experiment by varying the X-ray power and the flood gun parameters, in order to identify a region of the parameter space where reproducible binding energies are obtained (i.e. where the charging effects are fully compensated), by taking as a guideline the C 1s energy of inherent contaminants, which must be obtained at 284.60 ± 0.05 eV.

### Electrical measurements

A complex set-up was used for measurements, comprising off: a Lakeshore cryoprober model CPX-VF, a ferroelectric tester model TF2000 from aixACCT (for hysteresis loops), and an impedance analyzer model HP 4194A (for C-V characteristics) and a Keithley 6517 electrometer (for I-V characteristics). Hysteresis measurements were performed using a triangular voltage wave with 1 kHz frequency. The capacitance measurements were performed using an a.c. small signal of 0.1 V amplitude and 100 kHz frequency. I-V measurements were performed using hysteresis type measurements (zero-(+Vmax)-zero-(−Vmax)-zero). Only the sweeping down part from Vmax to zero was further considered^62^.

## Additional Information

**How to cite this article**: Pintilie, L. *et al.* Polarization induced self-doping in epitaxial Pb(Zr_0.20_Ti_0.80_)O_3_ thin films. *Sci. Rep.* **5**, 14974; doi: 10.1038/srep14974 (2015).

## Supplementary Material

Supplementary Information
