# Supplementary material for: Polarization induced self-doping in epitaxial Pb(Zr0.20Ti0.80)O3 thin films
Source: Sci Rep. 2015 Oct 8;5:14974. doi: 10.1038/srep14974 (PMC4597211; doi:10.1038/srep14974)
Supplement: Supplementary Information [file srep14974-s1.doc]

**Supplemental information**

**Polarization induced self-doping in epitaxial Pb(Zr,Ti)O_3_ thin films**

Lucian Pintilie*, Corneliu Ghica, Cristian Mihail Teodorescu, Ioana Pintilie, Cristina Chirila, Iuliana Pasuk, Lucian Trupina, Luminita Hrib, Andra Georgia Boni, Nicoleta Apostol, Laura Abramiuc, Raluca Negrea, Mariana Stefan, Daniela Ghica

National Institute of Materials Physics, Atomistilor 105 bis, Magurele, 077125, Romania

*E-mail: pintilie@infim.ro

*EPR investigations*

The PZT powder grated from the PLD target was measured in the X-band at room temperature (RT) and at several temperatures between RT and 20 K in the Q-band. The recorded spectra were obtained with 10 scans. The spectra of the identified impurities with electronic spin *S* were analyzed using a spin Hamiltonian of the form:

(SE1)

The first term represents the Zeeman electronic interaction, while the second and the third terms represent the interaction with the crystalline field with axial and rhombic symmetry, respectively. The simulation of the experimental spectra, using the specific parameters for each observed paramagnetic center, was performed with the EasySpin v.4.5 software.^S1^

In the X-band EPR spectrum from Fig. S1 two lines can be observed at ~ 120 mT ( *g* ~ 5.9) and 180 mT (*g* ~ 4.3), attributed to isolated Fe^3+^ ions with *S* = 5/2 [ref. 30 in the main text]. The lower field line belongs to tetragonal centers consisting of Fe^3+^ ions localized at B sites with an oxygen vacancy in the first coordination sphere. We have simulated their spectrum using the spin Hamiltonian parameters of the (Fe’_Ti_ – V_O_^**^)^*^ centers observed in PbTiO_3_, namely *g* = 2.002, *B*_2_^0^ = 11.76 GHz and a peak-to peak linewidth Δ*B* = 4 mT [ref. 31 in the main text]. The *g* = 4.3 line is characteristic to isolated Fe^3+^ ions in a rhombic environment.


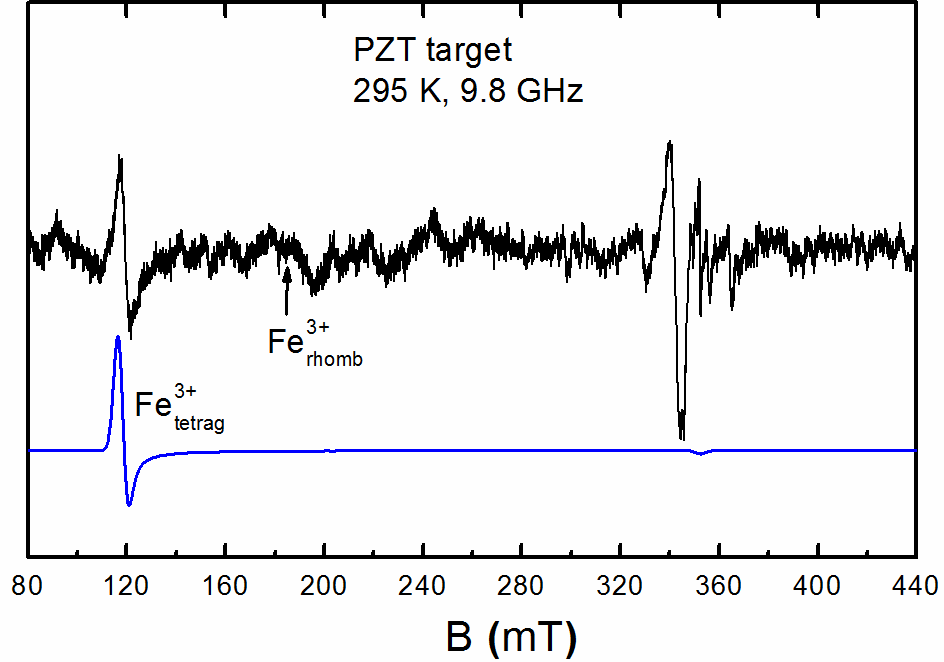


Fig. S1. X-band EPR spectrum of the PZT powder grated from the PLD target (black line). Blue line: simulation of a tetragonal Fe^3+^ spectrum with the EPR parameters of the (Fe’_Ti_ – V_O_^**^)^*^ centers in PbTiO_3_.


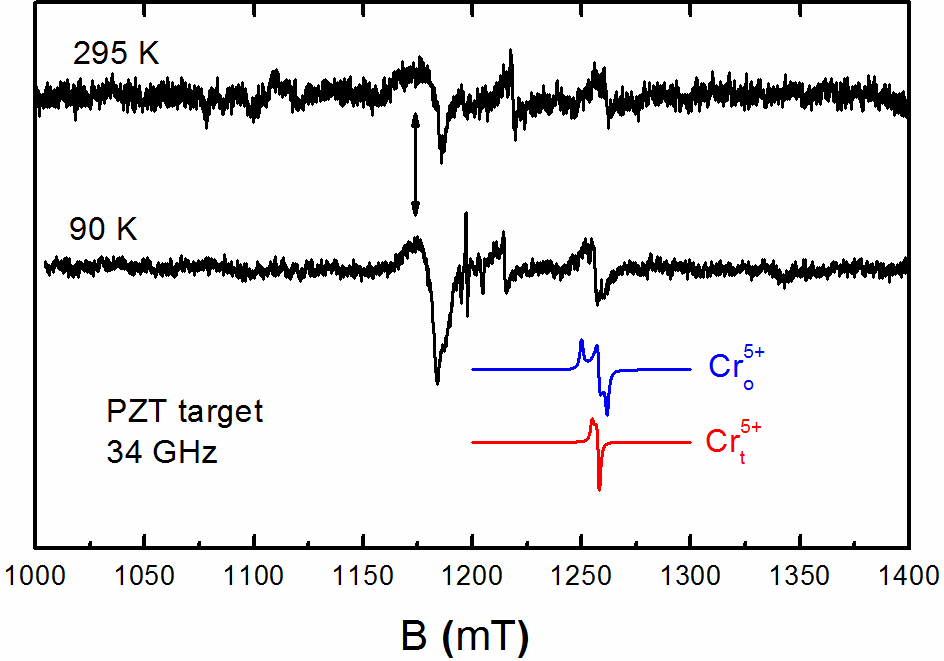


Fig. S2. Q-band EPR spectra measured at RT and 90 K on the PZT powder grated from the PLD target (black line). Blue and red lines: simulated spectra with the EPR parameters of the Cr^5+^ centers in PbTiO_3_. Arrows: background signal.

Figure S2 displays a detailed view of the higher field region of the EPR spectra measured at RT and 90 K. New lines become visible in the 90 K spectrum, especially in the *g* ~2 region. As seen in the figure, the complex signal at ~1260 mT is similar to the spectra reported for two Cr^5+^ (*S* = 1/2) paramagnetic centers in PbTiO_3_ [ref. 32 in the text], one with orthorhombic symmetry Cr^5+^_o_ and one with tetragonal symmetry Cr^5+^_t_. The spectra of these centers were simulated with the EPR parameters given in ref. [32], namely *g*_x_ = 1.930; *g*_y_ = 1.936; *g*_z_ = 1.9483 for Cr^5+^_o_ and *g*_x_ = *g*_y_ = 1.936; *g*_z_ = 1.9408 for Cr^5+^_t_. The EPR lines observed in the magnetic field range of 1170 – 1220 mT are very similar to the spectra from intrinsic defects observed in TiO_2_ [ref. 33 in the text] and BaTiO_3_ [ref. 34 in the text], associated with F-centers, oxygen centered radicals or Ti/Zr vacancies, and therefore we attribute them the same origin.

The lower field Q-band spectra (50-1000 mT) showed only the isolated Fe^3+^ signals for all investigated temperatures. No new spectra which could be attributed to the presence of other paramagnetic impurities were observed down to 20 K.


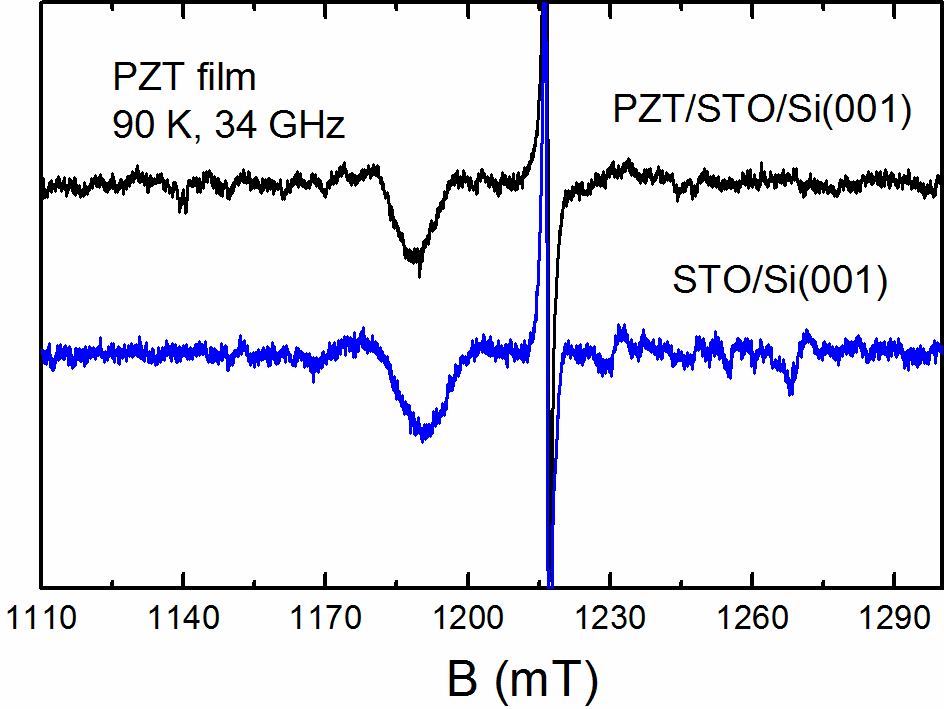


Fig. S3. Q-band EPR spectra measured at 90 K on the STO/Si substrate (blue line) and on the PZT film deposited on such a substrate (black line).

In order to check the transfer of impurities from the target to the film, we have investigated a PZT film of 1 μm thickness, deposited on a highly resistive floating zone Si(001) substrate with a 40 nm buffer layer of SrTiO_3_ (STO). The EPR spectra were recorded with 10 mW microwave power, 4 G modulation amplitude and 15 scans. As one can see in Fig. S3, the spectra of the PZT/STO/Si film are similar with those of the reference STO/Si substrate. The only EPR lines observed at all temperatures in the whole available field range were the sharp signal at 1217 mT with *g* = 2.0056, from dangling-bonds in the Si substrate,^S2^ and the background signal at 1185 mT.

The impurity content in the PZT film is expected to be similar with the impurity content in the PZT powder extracted from the PLD target, both as impurity types and concentration. Considering the sensitivity of ~10^10^ spins/Gauss of the EPR spectrometers and the volume of the measured PZT film of ~10^-2^ mm^3^, we can estimate the concentration of eventual impurities in the film to be below the detection limit of ~10^22^ m^-3^. The concentration of Fe^3+^ and Cr^5+^ ions in the PZT powder extracted from the PLD target can be also estimated at a few ppm or ~10^22^ m^-3^. The amount of measured PZT powder was three orders of magnitude larger than the amount of PZT in the film sample, which explains why it was possible to observe the impurity spectra in the powder.

*Structural investigations*

**PZT/SRO/STO (5 nm PZT) sample**


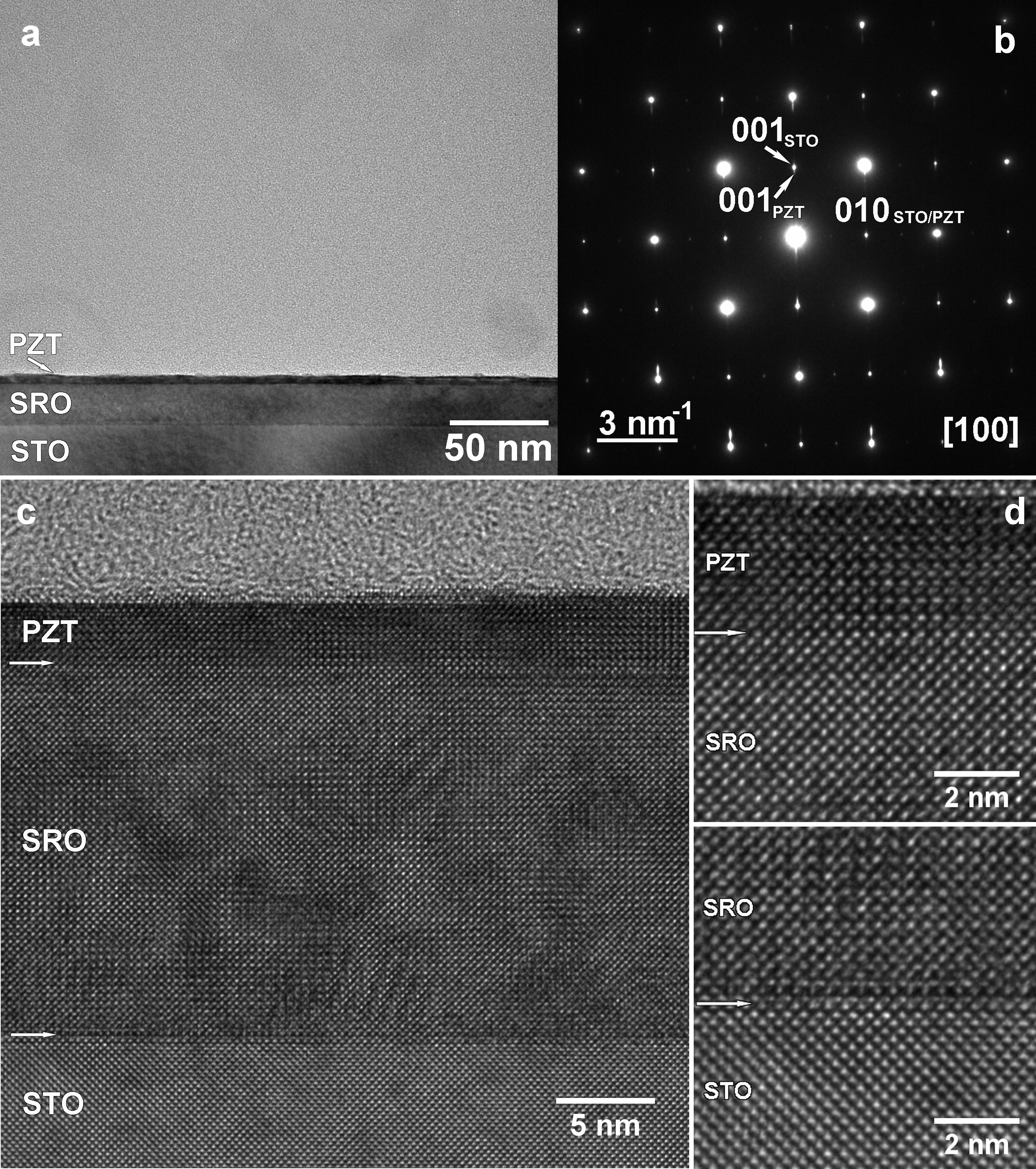


Fig. S4 (a) Low-magnification cross-section TEM image of the PZT/SRO/STO structure (5 nm PZT sample); (b) SAED pattern from the substrate and the deposited layers indicating epitaxial growth of the SRO and PZT layers; (c) low magnification HRTEM image of the PZT/SRO/STO structure confirming the epitaxial growth (white arrows indicate the SRO-STO and PZT-SRO interfaces); (d) HRTEM images at the PZT-SRO and SRO-STO interfaces (white arrows indicate the SRO-STO and PZT-SRO interfaces).

**PZT/SRO/STO (20 nm PZT) sample**

**
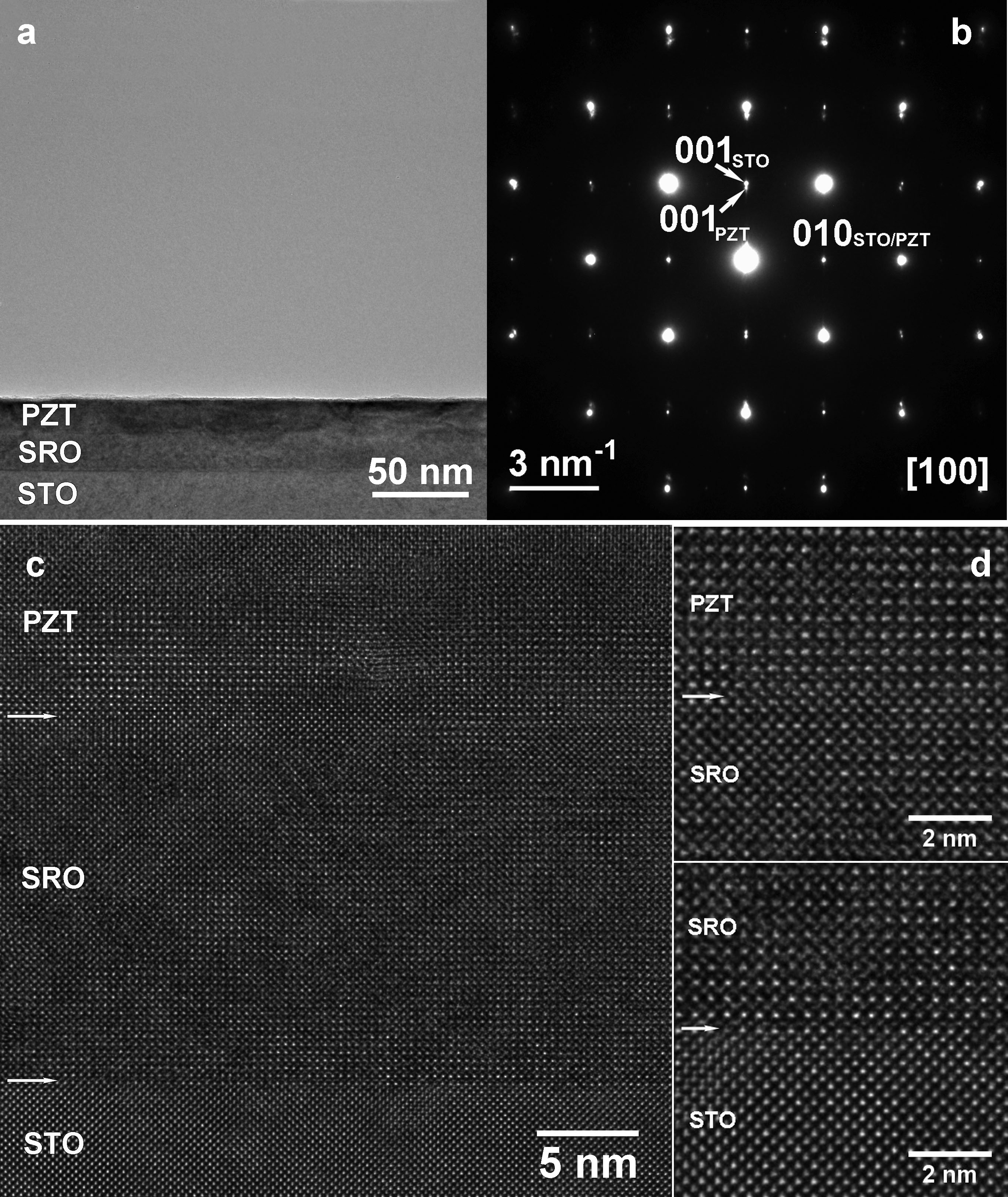
**

a

Fig. S5 (a) Low-magnification cross-section TEM image of the PZT/SRO/STO structure (20 nm PZT sample); (b) SAED pattern from the substrate and the deposited layers indicating epitaxial growth of the SRO and PZT layers; (c) low magnification HRTEM image of the PZT/SRO/STO structure confirming the epitaxial growth (white arrows indicate the SRO-STO and PZT-SRO interfaces); (d) HRTEM images at the PZT-SRO and SRO-STO interfaces (white arrows indicate the SRO-STO and PZT-SRO interfaces).

**PZT/SRO/STO (50 nm PZT) sample**


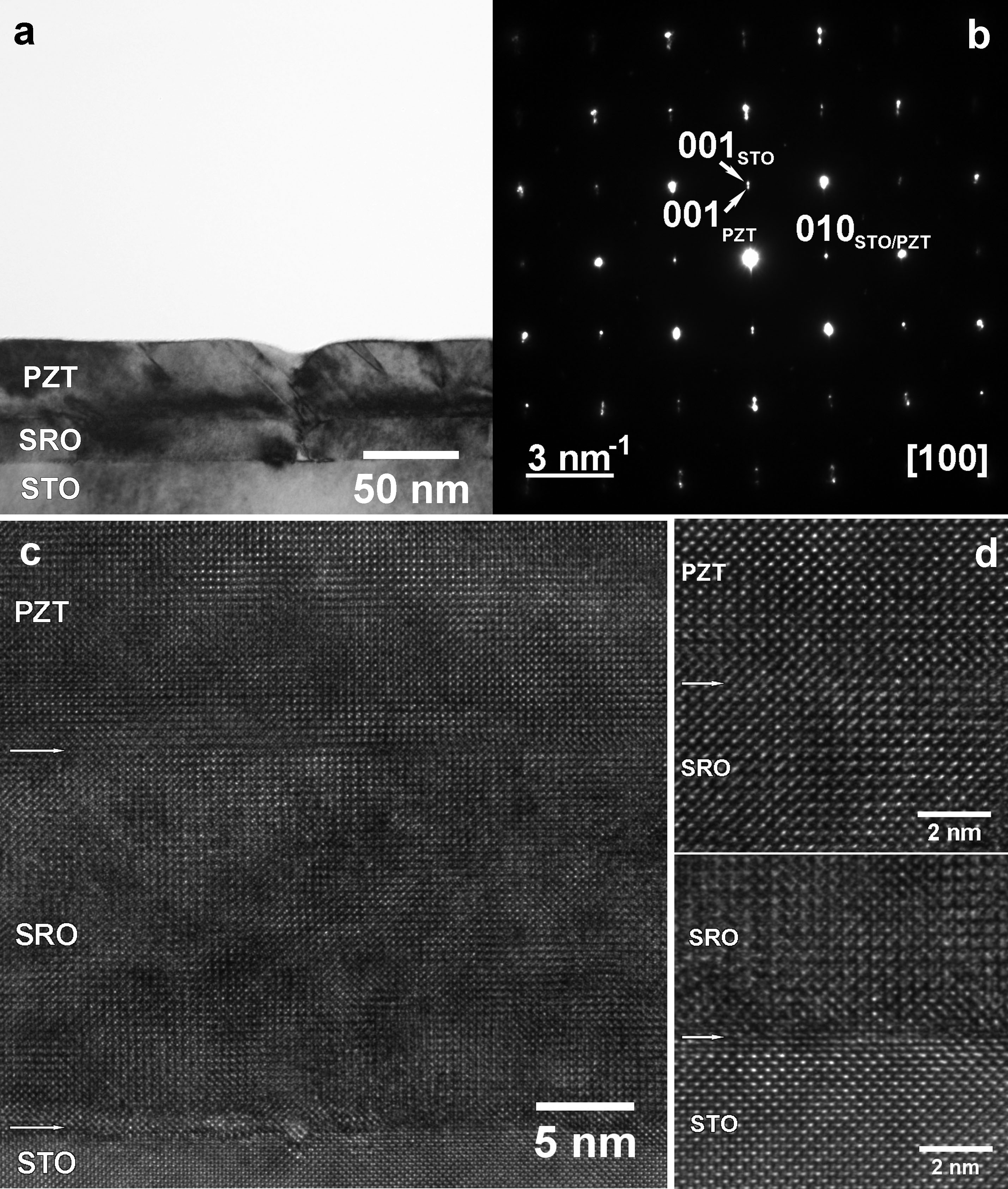


Fig. S6 (a) Low-magnification cross-section TEM image of the PZT/SRO/STO structure (50 nm PZT sample); (b) SAED pattern from the substrate and the deposited layers indicating epitaxial growth of the SRO and PZT layers; (c) low magnification HRTEM image of the PZT/SRO/STO structure confirming the epitaxial growth (white arrows indicate the SRO-STO and PZT-SRO interfaces); (d) HRTEM images at the PZT-SRO and SRO-STO interfaces (white arrows indicate the SRO-STO and PZT-SRO interfaces).

**PZT/SRO/STO (150 nm PZT) sample**


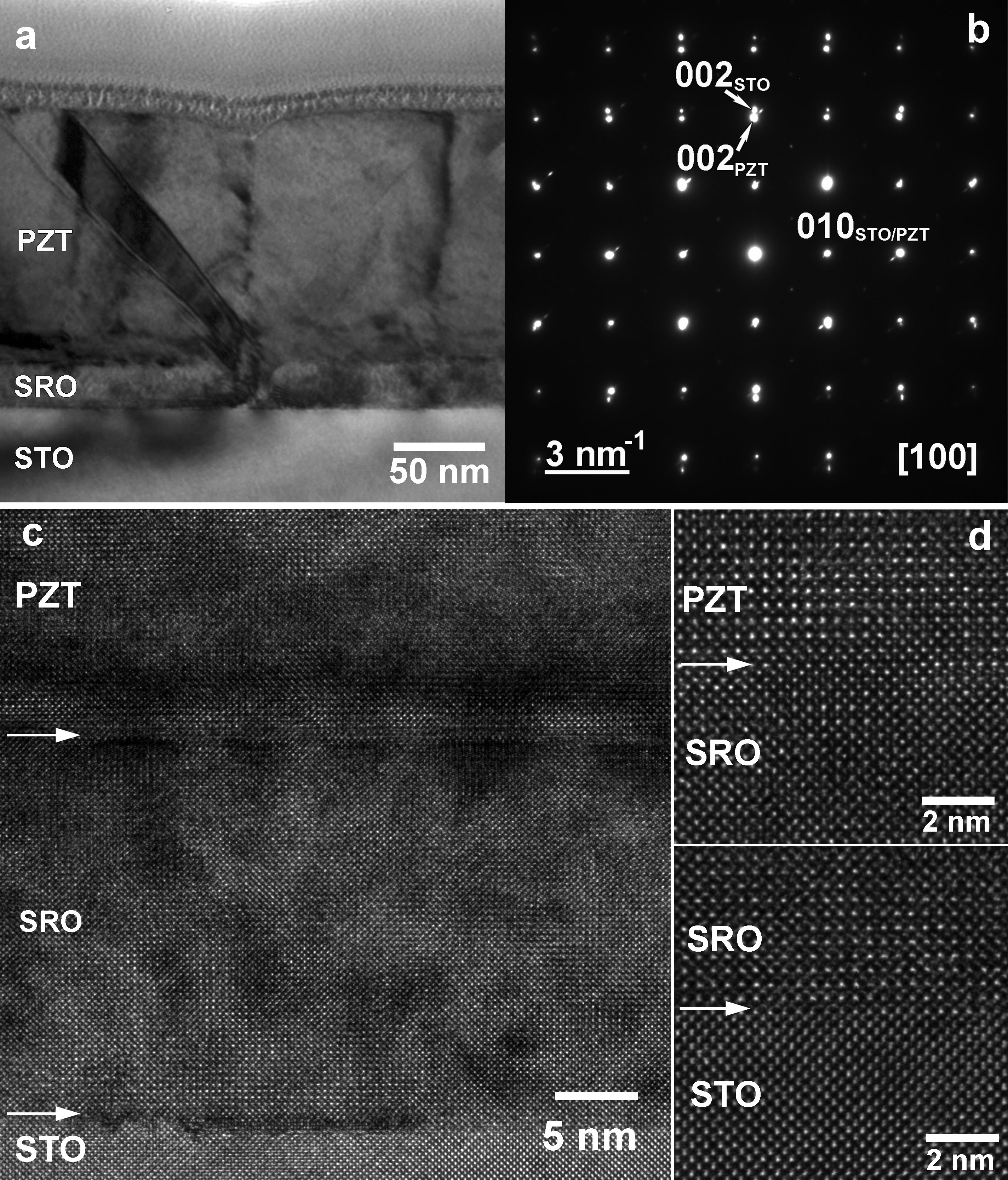


Fig. S7 (a) Low-magnification cross-section TEM image of the PZT/SRO/STO structure (150 nm PZT sample); (b) SAED pattern from the substrate and the deposited layers indicating epitaxial growth of the SRO and PZT layers; (c) low magnification HRTEM image of the PZT/SRO/STO structure confirming the epitaxial growth (white arrows indicate the SRO-STO and PZT-SRO interfaces); (d) HRTEM images at the PZT-SRO and SRO-STO interfaces (white arrows indicate the SRO-STO and PZT-SRO interfaces).

**PZT/SRO/STO (250 nm PZT) sample**


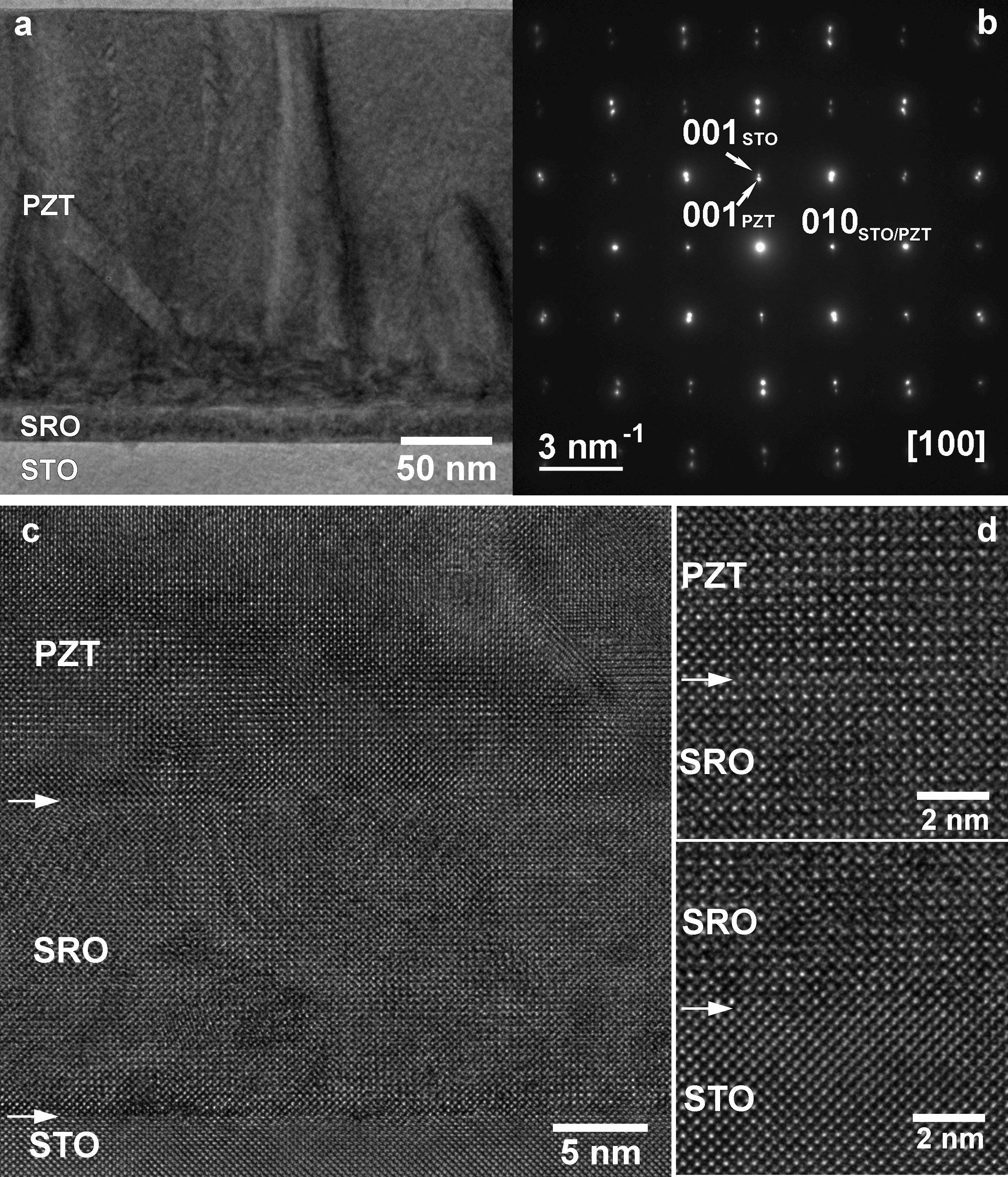


Fig. S8 (a) Low-magnification cross-section TEM image of the PZT/SRO/STO structure (250 nm PZT sample); (b) SAED pattern from the substrate and the deposited layers indicating epitaxial growth of the SRO and PZT layers; (c) low magnification HRTEM image of the PZT/SRO/STO structure confirming the epitaxial growth (white arrows indicate the SRO-STO and PZT-SRO interfaces); (d) HRTEM images at the PZT-SRO and SRO-STO interfaces (white arrows indicate the SRO-STO and PZT-SRO interfaces).

The analytical TEM investigations have been performed on a probe-corrected JEM ARM 200F analytical microscope. The cross-section specimens for TEM observations have been prepared by mechanical polishing followed by ion milling at low angle in a Gatan PIPS installation.

The TEM images (Fig. S4a, S5a, S6a, S7a, S8a) at low magnification show the morphology of the deposited layers and the thickness of the PZT thin film (5 nm, 20 nm, 50 nm, 150 nm and 250 nm). The dark contrast visible in the vicinity of the interfaces in PZT films is due to strain field. Ferroelectric domains have been observed in the PZT films with a thickness higher than of 50 nm.

The selected-area electron diffraction (SAED) patterns (Fig. S4b, S5b, S6b, S7b, S8b) indicate the epitaxial growth of the layers on the STO substrate and the tetragonality of the PZT thin film. The misfit between STO substrate and PZT thin film is evidenced by the 00l spots split. The interfaces between layers lead to an elongation of the diffraction spots in SAED pattern. The orientation relationship between the PZT thin film and the STO substrate is [001]_PZT_|| [001]_STO_ and [010]_PZT_||[010]_STO_.

HRTEM images (Fig. S4c, S5c, S6c, S7c, S8c) and the zoomed HRTEM images at the interfaces confirm the epitaxial growth and reveal in details the good quality of the PZT-SRO and SRO-STO interfaces.

*The C 1s XPS spectra*

The spectra (Fig. S9) are deconvoluted with Voigt singlets, whose assignment to C-related bonding states is attributed according to Ref. [45], and indicated on the graph. In the insert of Fig. S9 is represented the dependence of C=C, C-OH and O=C-O components with the PZT film thickness. It may be reasonably supposed that the amount of contaminants is roughly the same, irrespective on the film thickness. Therefore, the assumption from the main text that the O 1s component due to the C-OH contamination is constant for all films is reasonable.


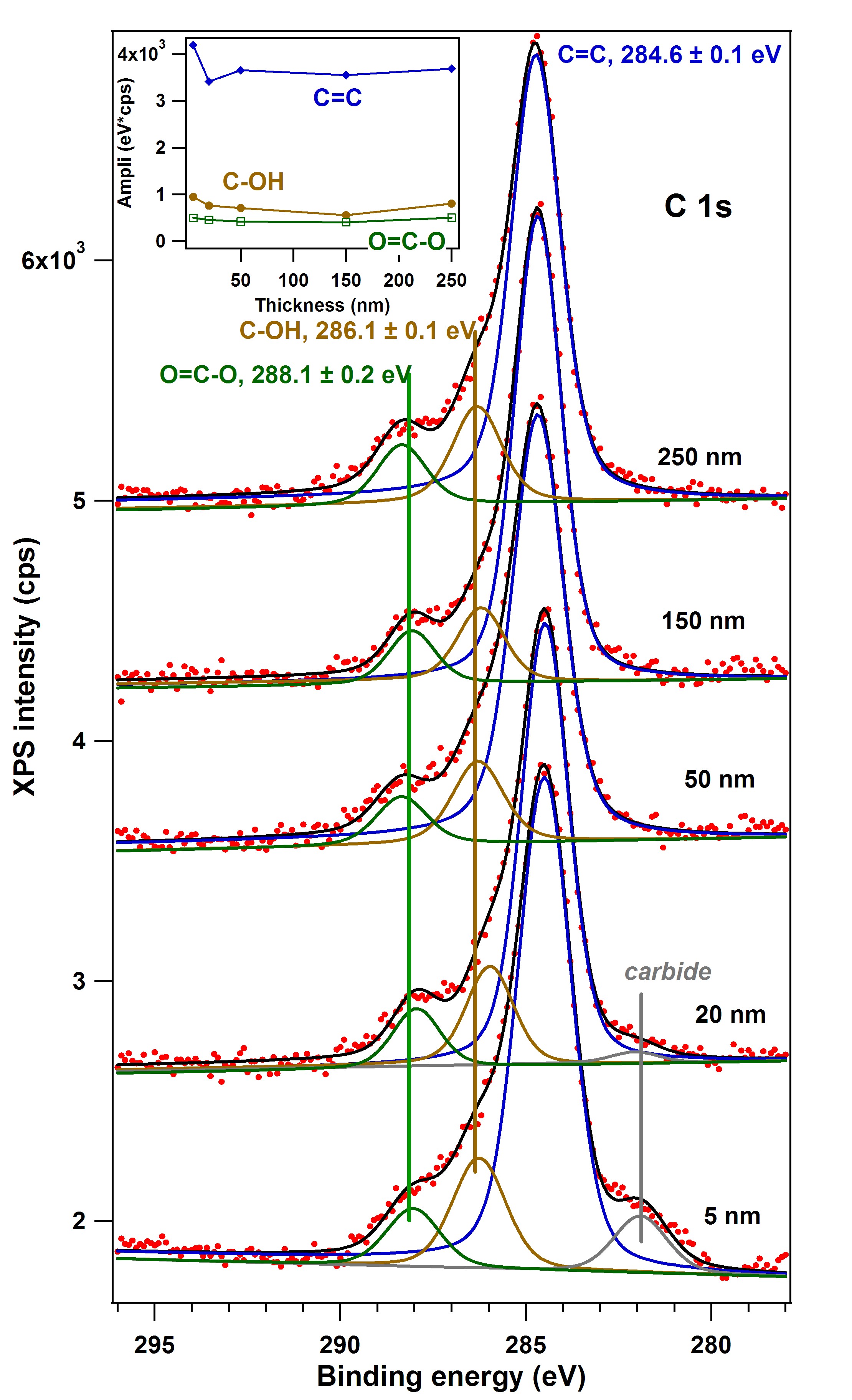


Fig. S9. The XPS spectra for C 1s. The inset shows the estimated amount of contaminants on the surface of the PZT films of different thicknesses.

*X-ray photoelectron spectroscopy data for 45° off-normal emission*


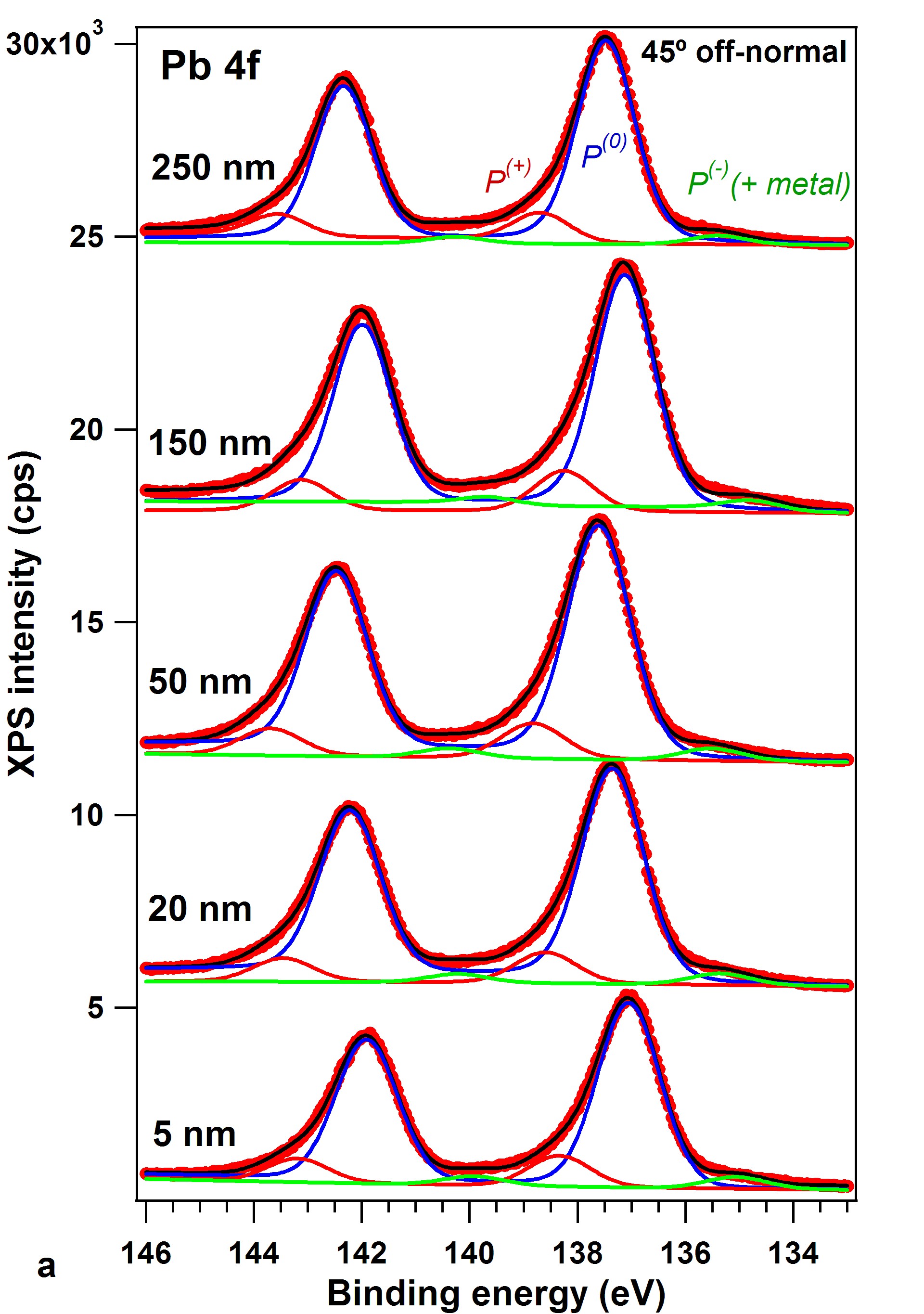

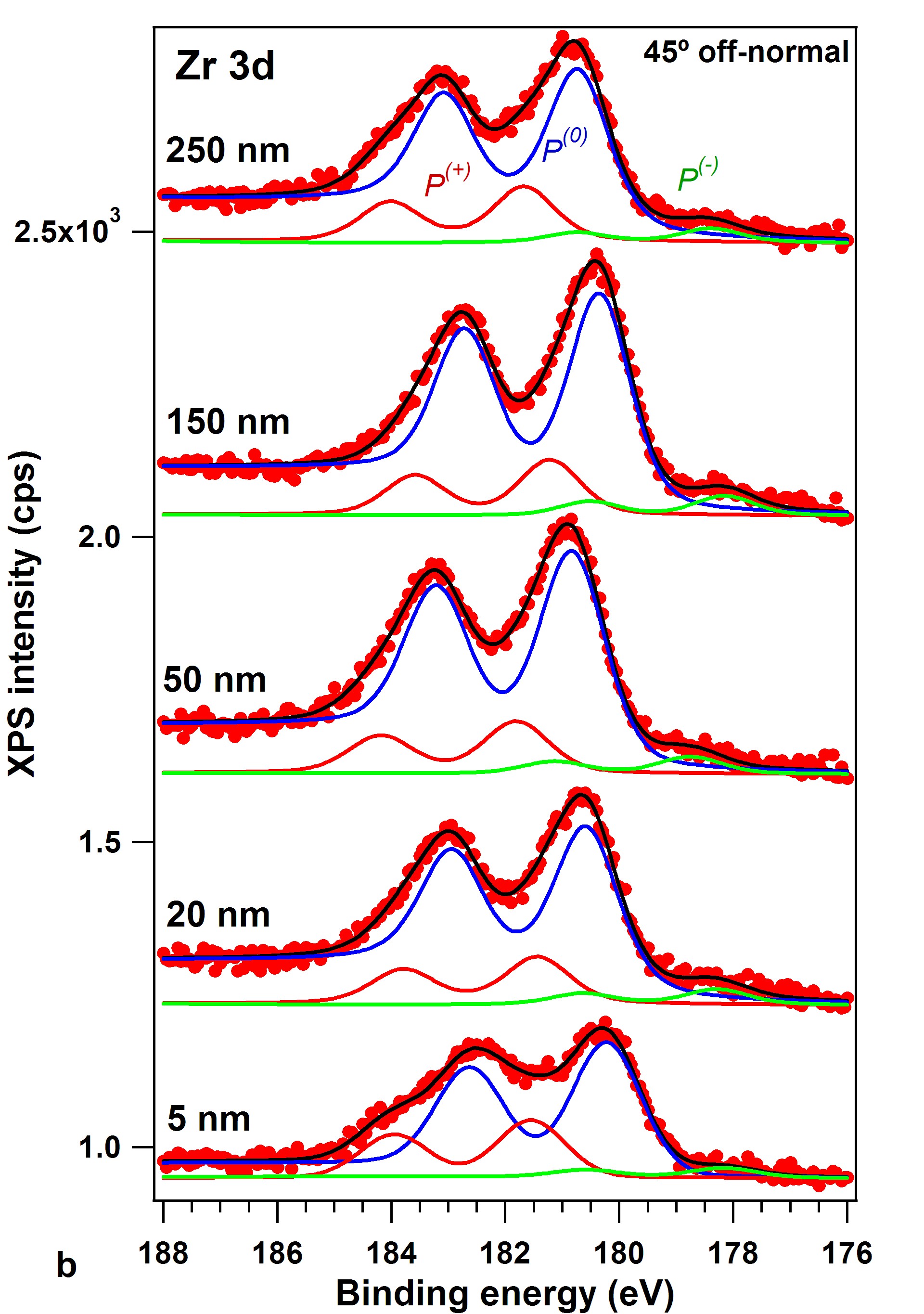


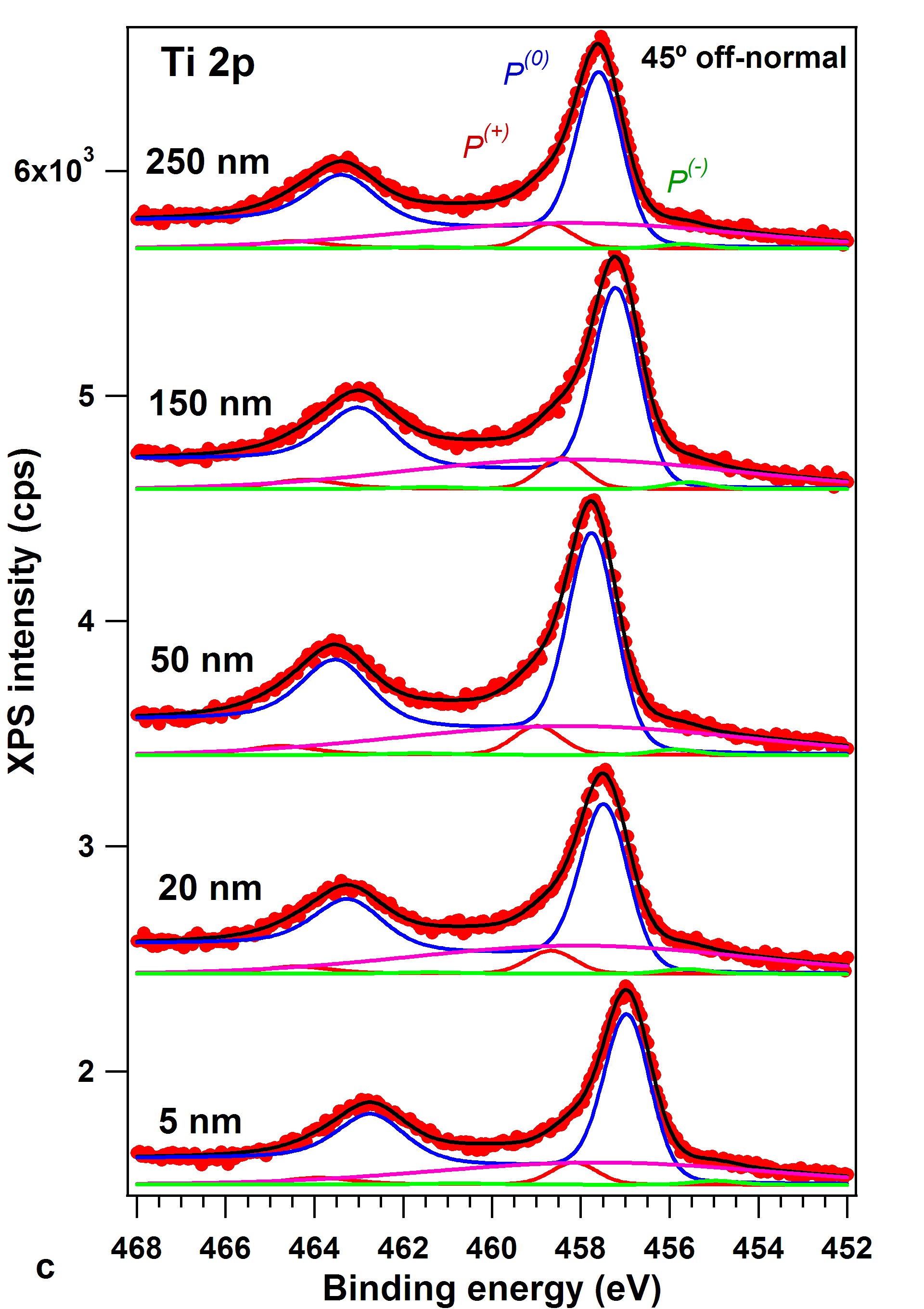

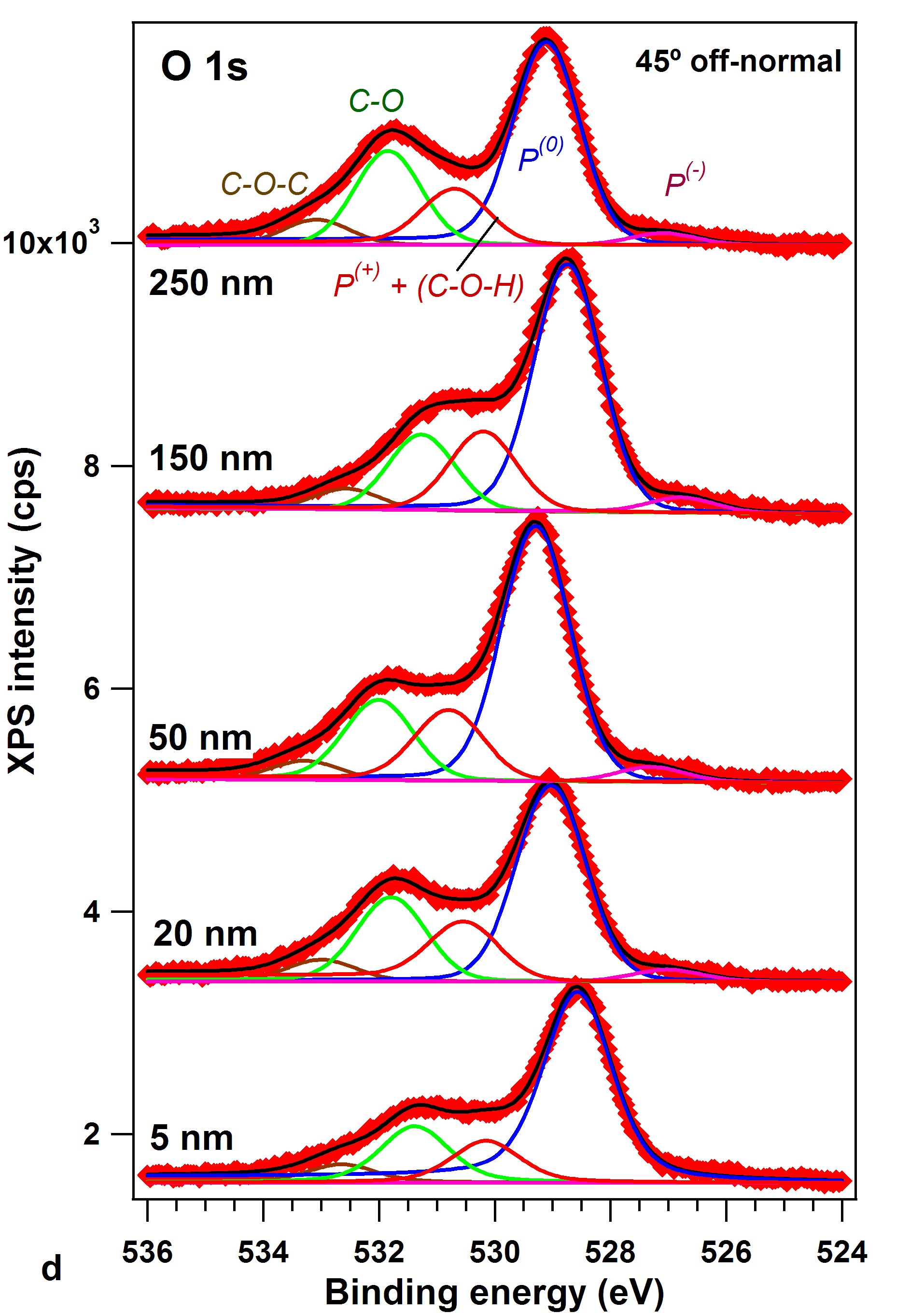


Fig. S10. Intensity ratios between the high or low binding energy components (P^(+)^ or P^(-)^) and the main component (P^(0)^) for the Pb 4f spectra, for normal emission and for 45° off-normal.

If the low binding energy component would have been due to metal Pb on the surface only, then at oblique emission its relative intensity with respect to the main component would have increased: we see from the figure below that this is not the case; on contrary, its relative intensity slightly decreases, with the exception of the film with lowest thickness. Also, the P^(+)^/P^(0)^ ratio is larger for the oblique emission by about 10-15 % for thicknesses above 20 nm (see Fig. S11). This can be regarded as a proof that at least some part of the P^(0)^ signal may be attributed to originate from the bulk (unbent regions). Another origin of this component may be due to the simultaneous effect of X-ray and electron irradiation (the latter produced by the flood gun), which might induce an over-compensation of the depolarization field for these areas (i.e. the surface charge density of the electron sheet near surface is larger than the absolute value of the polarization), such that the band bending is lost on these areas. See, for instance, Fig. 1(d) from Ref. [46].


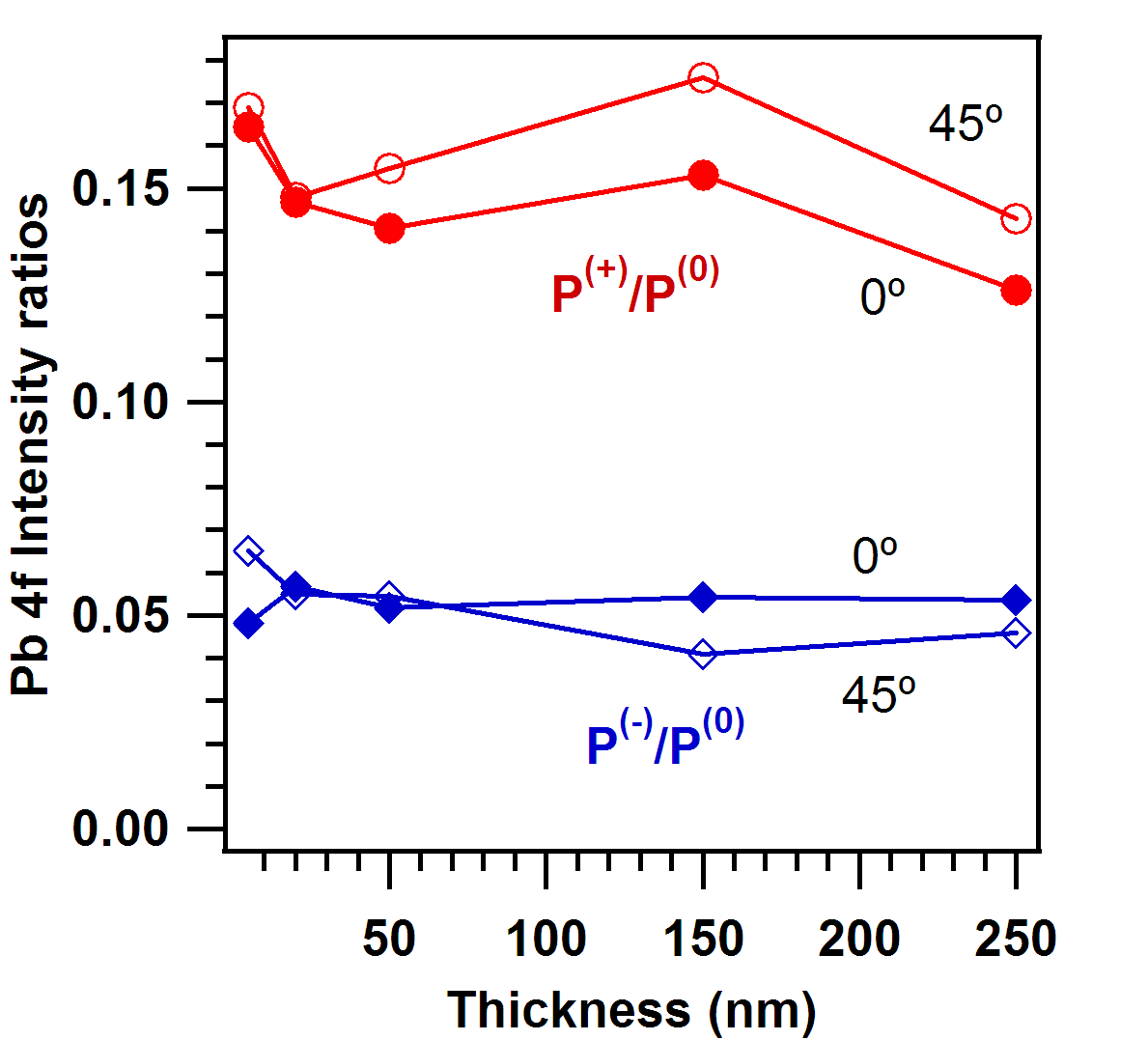


Fig. 11 P^(+)^/P^(0)^ and P^(-)^/P^(0)^ ratios for normal and oblique emissions.

*Electrical measurements*

- Hysteresis measurements and correction for the leakage current

In the case of the TF 2000 ferritester the hysteresis measurement is based on the integration of the current flowing through the ferroelectric capacitor. The total current and the corresponding integrated charge are given by:

$\begin{matrix} j(V)=j_{l}(V)+\frac{\partial D(V)}{\partial t} \\ Q(V)=\int j_{l}(V)dt+D(V) \end{matrix}$ (SE2)

Here j_l_ is the leakage current density, D is the electrical displacement, t is time and Q is the total integrated charge per unit area. $D=\varepsilon_{0}\varepsilon_{st}E+P_{S}$where ε_st_ is the static dielectric constant, E is the applied electric field and P_S_ is the spontaneous polarization in the ferroelectric material. Usually it is considered that the linear term $\varepsilon_{0}\varepsilon_{st}E$is much smaller than P_S_ thus D it is approximated with P_S_.

If the leakage current is negligible, then the integrated charge is equal with P_S_. If the leakage current is high, then it adds contribution to Q leading to inflated hysteresis loops, as shown in Fig.S10 for the PZT film of 10 nm thickness.

Fig. S10 Charge and current hysteresis for a 10 nm thick epitaxial PZT film

In figure S10 one can see that the current peaks associated to polarization switching are still visible in the current hysteresis. These peaks can be used to estimate the correct value for the polarization P_S_. For this purpose the current corresponding to sweep down voltages is extracted from the current corresponding to sweep up voltage for both polarities. In this way the contribution from the leakage current is subtracted from the total current and only the contribution from the polarization switching remains, as shown in Fig. S11.

Fig. S11 The current corresponding to the polarization switching, obtained after subtracting the leakage current from the total current (see also equation SE2).

By integrating the current in Fig. S11 one can obtain the correct value for P_S_ and to partly reconstruct the hysteresis loop for the ferroelectric polarization (see Fig. S12).

Fig. S12 The partly reconstructed hysteresis loop obtained by integrating the current peaks corresponding to polarization switching.

The polarization value is taken as the average of the values obtained by integrating the current peaks for the two voltage polarities in Fig. S12. The obtained value is of about 0.95 Cm^-2^. The same procedure was applied for all the samples and the corrected polarization value is around 100 Cm^-2^ in all cases (+/-0.05 Cm^-2^).

- Evaluation of the free carrier concentration

The carrier concentrations in PZT layers were determined in the same way as in the case of semiconductor Schottky contacts, using the following equation:

$n(V)=\frac{2}{q\varepsilon_{0}\varepsilon_{st}\left[d\left(1/C^{2})/dV\right)\right]}$ (SE3)

**References**

#### S1. Stoll, S. & Schweiger, A. EasySpin, a comprehensive software package for spectral simulation and analysis in EPR. *J. Magn. Res.* 178, 42-55 (2006).

S2. Ghica, C., Nistor, L. C., Stefan, M., Ghica, D., Mironov, B., Vizireanu, S., Moldovan, A. & Dinescu, M. Specificity of defects induced in silicon by RF-plasma hydrogenation. *Appl. Phys. A* **98**, 777–785 (2010).
